# Direct Synthesis of Allylic Sulfones via Hydrosulfonylation of 1,3-Dienes with Sulfinic Acids

**DOI:** 10.3390/molecules30081785

**Published:** 2025-04-16

**Authors:** Ke Guo, Shuaichen Zhang, Jing Zhang, Yu Ren, Xiaoqiang Chang, Peng Sun

**Affiliations:** 1 State Key Laboratory for Quality Ensurance and Sustainable Use of Daodi Herbs, Institute of Chinese Materia Medica, Academy of Chinese Medical Sciences, Beijing 100700, China; gk18337455008@163.com (K.G.); zhangsc664681@163.com (S.Z.); jzhang1227@icmm.ac.cn (J.Z.); 2 Artemisinin Research Center, Academy of Chinese Medical Sciences, Beijing 100700, China; 3 Xiyuan Hospital, China Academy of Chinese Medical Sciences, No.1 Xiyuan Playground, Beijing 100091, China; robinrenyu@foxmail.com; 4 School of Pharmacy, Bengbu Medical University, Bengbu 233000, China

**Keywords:** catalyst free, allylic sulfones, hydrosulfonylation, green chemistry, room temperature

## Abstract

Catalyst- and additive-free reactions for synthesizing valuable organic compounds have garnered significant attention in the context of sustainable development. As crucial structural motifs, allylic sulfones find extensive applications in pharmaceutical development and organic synthesis. Despite remarkable advances in allylic sulfone construction, catalyst-free and additive-free methodologies remain an underexplored frontier. Herein, we present an environmentally benign and atom-economical approach for synthesizing allylic sulfones by reacting electron-rich aryl-1,3-dienes with sulfinic acids, achieving yields of 10–94%. This transformation proceeds under ambient air at room temperature, eliminating the need for catalysts or additives. The protocol demonstrates exceptional regio- and chemo-selectivity, streamlined operational simplicity, and excellent scalability potential. This methodology establishes a sustainable and cost-effective paradigm for allylic sulfone synthesis, aligning with green chemistry principles.

## 1. Introduction

**Figure 1:**
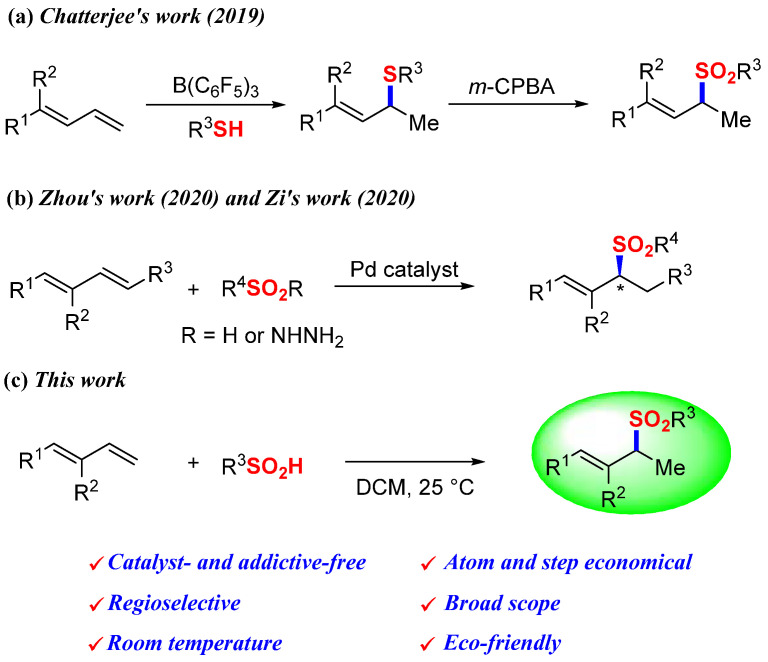
Hydrofunctionalization of 1,3-dienes to synthesize allylic sulfones: (**a**) tris(Pentafluorophenyl)borane-catalyzed hydrothiolation of 1,3-dienes [29]; (**b**) palladium-catalyzed hydrosulfonylation of 1,3-dienes [25,27]; (**c**) catalyst-free hydrosulfonylation of 1,3-dienes.

The pursuit of green chemistry has become a critical societal imperative, as chemical processes increasingly contribute to environmental pollution and hazardous waste accumulation [1,2,3,4]. Within this context, catalyst- and additive-free organic synthesis emerges as a transformative strategy that combines economic viability with environmental sustainability, particularly through its tolerance to ambient conditions and operational simplicity [1,4,5,6,7,8,9]. Allylic sulfones represent a privileged class of compounds extensively employed in pharmaceutical synthesis and materials science, owing to their versatile alkene functionality and stereochemical complexity [10,11,12,13]. This widespread utility has driven substantial research efforts toward developing efficient synthetic methodologies [14,15,16,17]. Conventional approaches—including transition-metal-catalyzed cross-coupling reactions—while effective, face practical limitations in industrial applications due to their reliance on costly noble metal catalysts and the generation of toxic byproducts [18,19,20,21,22,23,24,25,26,27]. These drawbacks not only increase production costs but also raise environmental and safety concerns associated with metal leaching and waste management. Recent advances in 1,3-diene hydrofunctionalization have opened new avenues for allyl compound synthesis [28,29,30,31,32,33]. Notable developments include a hydrothiolation protocol yielding 1,2-Markovnikov sulfides, explored by Chatterjee in 2019 [29], and palladium-catalyzed hydrosulfonylation strategies achieving chiral allylic sulfones with exceptional stereochemical control, reported by Zhou [27] and Zi [25] in 2020 (Figure 1a,b). However, the persistent requirement for metal catalysts in these systems underscores the need for more sustainable alternatives. In 2023, we explored the boron-catalyzed hydrosulfonylation of aryl 1,3-dienes, affording a broad scope of allylic sulfones in good yields [34]. Building on our foundational work in sulfone synthesis [9,34,35,36], herein, we report the first catalyst-free hydrosulfonylation between electron-rich 1,3-dienes (specifically substituted styrene derivatives) and sulfinic acids (Figure 1c). Although excessive amounts of 1,3-dienes, limited sulfonation reagent, and dichloromethane solvent are used, this pioneering procedure combines unprecedented operational simplicity with outstanding atom economy and regioselectivity, establishing a new benchmark for environmentally benign allylic sulfone production.

## 2. Results

Optimization of the Reaction Conditions. The model reaction between 1-(buta-1,3-dien-1-yl)-4-methoxybenzene (**1a**) and 4-methylbenzenesulfinic acid (**2a**) was systematically investigated (Table 1). Following extensive optimization, the allylic sulfone **3aa** was obtained in a 94% isolated yield with complete regiocontrol using dichloromethane (DCM) as a solvent under ambient conditions (25 °C, 8 h) (Entry **1**). Intriguingly, introducing BF_3_·OEt_2_ as a Lewis acid catalyst resulted in markedly diminished yield (Entry **2**), underscoring the self-sufficiency of the catalyst-free system. A control experiment indicated that **1a** disappeared in 30 min after the addition of BF_3_·OEt_2_. Solvent screening revealed that DCM is appropriate for this system, with polar aprotic solvents (Entries **3–6**) and ethereal solvents (Entries **7–11**) exhibiting substantially reduced efficiency (28–78% yields). Thermal analysis demonstrated inverse temperature dependence, where elevated temperatures at 40 °C and 70 °C diminished yields to 69% and 56%, respectively (Entries **12–13**). Stoichiometric studies indicated optimal performance at a 1:1 molar ratio (Entry **1**), with excess sulfinic acid (1.2–1.5 equiv.) failing to enhance productivity (<90% yields; Entries **14–15**). Notably, extending the reaction duration to 12 h provided no obvious improvement (Entry **1** vs. standard conditions).

Substrate Scope Evaluation. With optimal conditions established, we systematically evaluated the reaction generality through comprehensive substrate screening (Table 2). The protocol demonstrated broad compatibility with diverse sulfinic acids. Phenylsulfinic acid delivered adduct **3ab** in a 90% yield. Para-substituted aryl variants exhibited notable efficiency: chloro and bromo substituents afforded **3ad** (76%) and **3ae** (94%), respectively. Electron-deficient arenes bearing -CN, -CF_3_, and -NO_2_ groups proved competent, generating **3ac**, **3af**, and **3ag** in 62–81% yields. Polyhalogenated substrate **3ah** was obtained in a 79% yield, while meta- and ortho-substituted methyl/trifluoromethyl analogs (**3ai**–**3al**) achieved 64–83% efficiencies. The methodology extended to 2-naphthylsulfinic acid (81%, **3am**) and aliphatic derivatives (68%, **3an**), underscoring remarkable functional group tolerance. The structural diversification of dienes revealed critical electronic effects. Para-methyl substitution on the aryl diene (**1b**) yielded **3ba** (54%), whereas methoxy group removal (**1c**) drastically reduced efficiency to 10% (**3ca**). Meta- and ortho-substituted variants (**1d**–**1e**) provided **3da**–**3ea** in 24–27% yields, highlighting the essential role of para-electron donation. Naphthalene-containing diene 1f demonstrated good compatibility (67%, **3fa**). Notably, an electron-rich heteroaromatic system including thiophene and furan derivatives produced **3ha**–**3ja** with 61–72% efficiencies, establishing the method’s versatility in handling π-rich systems. The observed yield variations (10–94%) correlate strongly with the substituent electronic nature and positioning, where electron-donating groups at the diene’s para-position markedly enhance reactivity. This electronic sensitivity profile aligns with proposed mechanistic pathways involving charge-stabilized intermediates.

Calculable green metrics have been effectively employed as measurable tools for evaluating the eco-friendliness of reactions in the chemical and pharmaceutical industries [37]. Herein, the atom economy (AE) and environmental factor (E factor) of the processes were calculated to assess their green features, as shown in Table 3. Compared with the previous two methods developed by Zhou and Zi, this protocol exhibits a superior atom economy and E factor, indicating this is a promising solution for preparing allylic sulfones from the green chemistry perspective.

**Figure 2:**
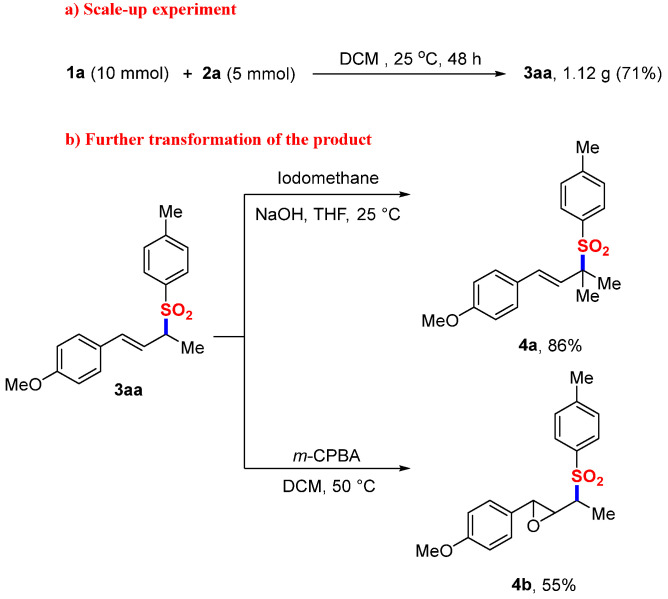
Further transformation of the generated product.

Synthetic Utility and Functionalization. To demonstrate industrial viability, scaled-up experiments (5 mmol) delivered allylic sulfone **3aa** through gram-scale synthesis (1.32 g, 71%), confirming operational robustness. The synthetic versatility of **3aa** was further exemplified through sequential derivatization (Figure 2): (1) base-mediated alkylation with NaOH/CH_3_I yielded tertiary sulfone **4a** (86%), and (2) epoxide installation employing m-chloroperbenzoic acid (m-CPBA) afforded β-epoxy-sulfone **4b** (55%), a privileged scaffold in bioactive molecule synthesis [38,39,40,41]. Remarkably, **4a** served as a competent electrophile in nickel-catalyzed Suzuki–Miyaura cross-couplings [42], enabling the modular construction of polyfunctional sulfone architectures. This cascading functionalization strategy establishes **3aa** as a versatile linchpin intermediate for synthesis.

**Figure 3:**
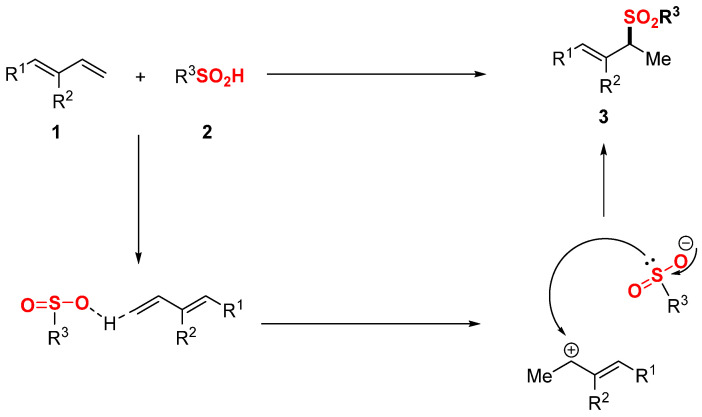
Proposed reaction pathway.

Based on previous reports and initial experimental findings [43,44,45], a proposed mechanism for this reaction is presented in Figure 3. The hydrosulfonylation of 1,3-dienes 1 with sulfinic acid **2** is proposed to proceed through the following steps: (1) proton migration from **2** to the terminal of 1,3-diene generates an allylic carbocation intermediate; (2) the benzenesulfonic acid anion acts as a nucleophile and attacks the allylic carbocation, forming product **3**.

## 3. Materials and Methods

General information: All commercially available solvents and reagents were used without further purification. The 1,3-dienes and sulfinic acids employed were synthesized according to the literature [46,47,48]. Thin-layer chromatography (TLC) employed glass 0.25 mm silica gel plates. Flash column chromatography was carried out using commercially available 200–300 mesh under pressure unless otherwise indicated. Gradient flash chromatography was employed, eluting with PE/EA and listed as volume/volume ratios. ^1^H and ^13^C NMR spectra were collected with a BRUKER AV-600 (600 MHz) spectrometer using CDCl_3_ as solvent. Chemical shifts in ^1^H NMR were recorded in parts per million (ppm, *δ*) relative to tetramethylsilane (*δ* = 0.00 ppm), with the solvent resonance as the internal standard (CDCl_3_: *δ* = 7.26 ppm). Data are reported as follows: chemical shift in ppm (*δ*), multiplicity (s = singlet, d = doublet, t = triplet, q = quartet, m = multiplet), coupling constant (Hz), and integration. Chemical shifts in ^13^C NMR are reported in ppm with the solvent as the internal standard. High-resolution mass measurement was performed using a Waters Q-TOF 6520 mass spectrometer with electron spray ionization (ESI) as the ion source. Melting point (m.p.) was measured on a microscopic melting point apparatus.

General procedure for the model reaction: In total, 0.20 mmol of 1,3-diene (2 equiv.) and 0.10 mmol of sulfinic acid (1 equiv.) were added to a 25 mL round-bottom flask, followed by the addition of 3.0 mL of DCM. The mixture was stirred at room temperature for 8 h. Upon completing the reaction, it was quenched with saturated ammonium chloride solution, and the organic phase was sequentially washed with water and saturated sodium chloride solution. The solvent was then removed under reduced pressure. The resulting crude product was purified via flash chromatography on silica gel using petroleum ether and ethyl acetate as eluents in a PE/EA ratio ranging from 10:1 to 4:1.

## 4. Conclusions

We have presented the first catalyst-/additive-free hydrosulfonylation of 1,3-dienes with sulfinic acids that proceeds with high regioselectivity under ambient conditions. This operationally simple protocol demonstrates exceptional atom economy (calculated at 95%) and broad functional group tolerance across electronically diverse substrates, providing a robust platform for streamlined allylic sulfone construction. At present, the scope of this reaction is limited to electron-rich aryl 1,3-dienes without a substituent at the terminal site of the diene. Our ongoing research aims to further elucidate the mechanistic details and extend the methodology to stereoselective variants of medicinally relevant sulfone derivatives.

## Figures and Tables

**Table 1:** Optimization of the conditions.

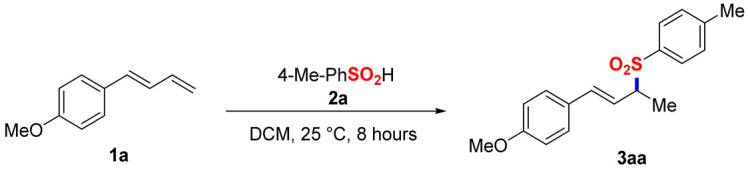		
**Entry**	**Variation from the Standard Conditions ^a^**	**Yield ^b^**
1	None	94% (93%) ^c^
2	Added 10 mol% BF_3_·OEt_2_	35%
3	With petroleum ether instead of DCM	32%
4	With CYH instead of DCM	25%
5	With toluene instead of DCM	30%
6	With THF instead of DCM	0
7	With EA instead of DCM	0
8	With acetone instead of DCM	0
9	With MeOH instead of DCM	0
10	With MeCN instead of DCM	0
11	With MTBE instead of DCM	0
12	40 °C	69%
13	70 °C	56%
14	**1a**/**2a** = 1.2	67%
15	**1a**/**2a** = 1.5	83%

^a^ Standard reaction conditions: **1a** (0.20 mmol, 2 equiv.), **2a** (0.10 mmol, 1 equiv.), DCM (3 mL), 25 °C, and 8 h. ^b^ Isolated yield. ^c^ 12 h.

**Table 2:** Scope of the reaction ^a,b^.

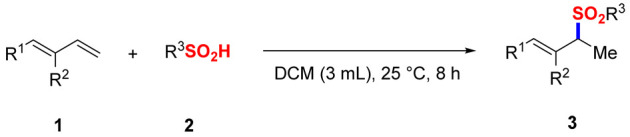
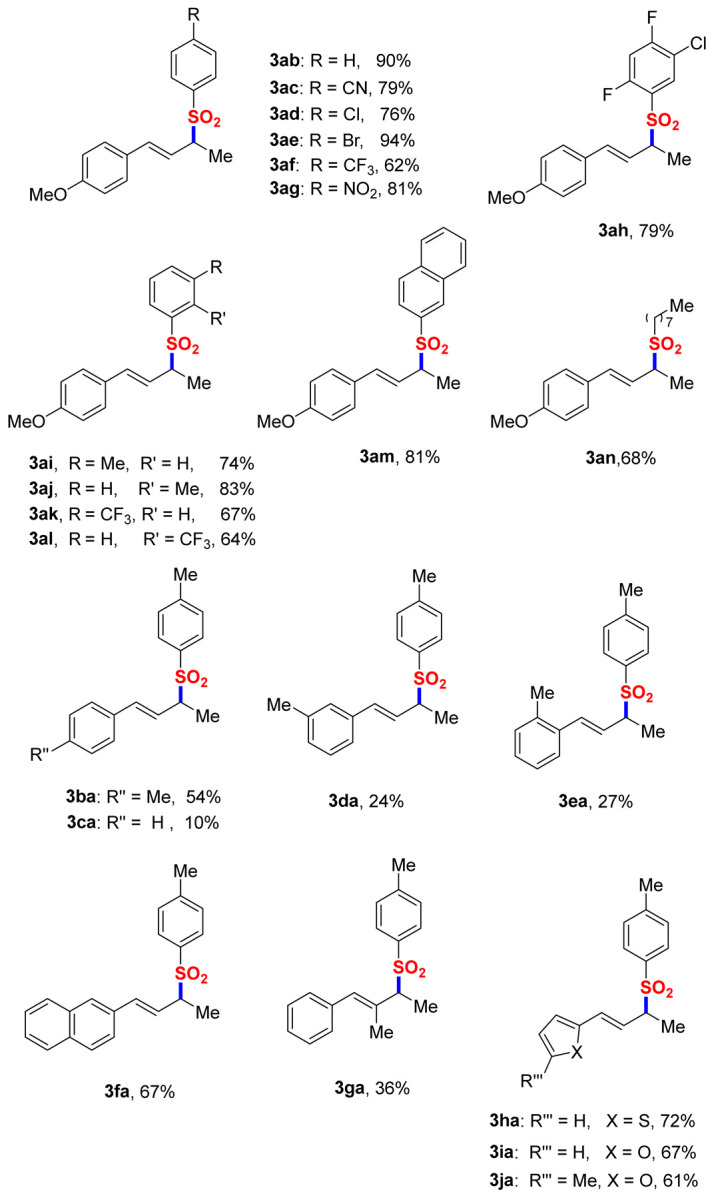

^a^ Standard reaction conditions: **1** (0.20 mmol, 2 equiv.), **2** (0.10 mmol, 1 equiv.), DCM (3 mL), 25 °C, and 8 h. ^b^ Isolated yield.

**Table 3:** Green chemistry metrics of previous reports and our method.

	Atom Economy (AE)	Environmental Factor (E Factor)
Zhou’s work [27]	0.83	1.28
Zi’s work [25]	0.91	0.65
Our work	0.95	0.60

## Data Availability

All relevant data are within the manuscript/Supplementary Materials.

## Supplementary Materials

The following supporting information can be downloaded at: https://www.mdpi.com/article/10.3390/molecules30081785/s1. Figure S1. Structural formulas of substrate 1,3-dienes 1. Figure S2. Structural formulas of substrate sulfinic acids 2. Figures S3–S27. ^1^H NMR and ^13^C NMR spectra of compounds **3aa**, **3ab**, **3ac**, **3ad**, **3ae**, **3af**, **3ag**, **3ah**, **3ai**, **3aj**, **3ak**, **3al**, **3am**, **3an**, **3ba**, **3ca**, **3da**, **3ea**, **3fa**, **3ga**, **3ha**, **3ia**, **3ja**, **4a**, **4b**.
