# Supplementary material for: Direct Synthesis of Allylic Sulfones via Hydrosulfonylation of 1,3-Dienes with Sulfinic Acids
Source: Molecules. 2025 Apr 16;30(8):1785. doi: 10.3390/molecules30081785 (PMC12029303; doi:10.3390/molecules30081785)
Supplement: Supplementary file 1 [file molecules-30-01785-s001.zip › molecules-3547252-supplementary.pdf]

Supporting Information

Direct Synthesis of Allylic Sulfones via Hydrosulfonylation of 1,3-Dienes with Sulfinic Acids

Ke Guo[1,2], Shuaichen Zhang[1,2], Jing Zhang[1,2], Yu Ren[3], Xiaoqiang Chang[4*], Peng Sun[1,2*]

1 State Key Laboratory for Quality Ensurance and Sustainable Use of Dao-di Herbs, Institute of Chinese Materia Medic, Academy of Chinese Medical Sciences, Beijing, 100700, China.

2 Artemisinin Research Center, Academy of Chinese Medical Sciences, Beijing, 100700, China

3 Xiyuan Hospital, China Academy of Chinese Medical Sciences, No.1 Xiyuan Playground, Beijing, 100091, China.

4 School of Pharmacy, Bengbu Medical University, Bengbu 233000, Anhui, China

* Correspondence: 15538186968@163.com (X. C.); psun@icmm.ac.cn (P. S.)

Table of Contents

1. General Information........2
2. Preparation of the Substrates........3
3. Experimental Procedure........5
4. ^{1}H and ^{13}C-NMR Spectra of the Title Compounds........13
5. References........43

1. General Information

Reagents and Solvents: CYH refers to Cyclohexane, *m*-CPBA refers to 3-chloroperoxybenzoic acid, PE refers to petroleum ether (b.p. 60-90 °C), DCM refers to dichloromethane, MTBE refers to methyl tert-butyl ether, EA refers to ethyl acetate, NaOH refers to Sodium hydroxide, KO^{t}Bu refers to potassium *t*-butoxide. Unless noted, commercially available reagents were used without further purification.

Chromatography: Thin layer chromatography (TLC) employed glass 0.25 mm silica gel plates. Flash column chromatography was carried out using commercially available 200-300 mesh under pressure unless otherwise indicated. Gradient flash chromatography was conducted eluting with PE/EA which are listed as volume/volume ratios.

Data collection: ^{1}H and ^{13}C NMR spectra were collected on BRUKER AV-600 (600 MHz) spectrometer using $CDCl_3$ as solvent. Chemical shifts of ^{1}H NMR were recorded in parts per million (ppm, δ) relative to tetramethylsilane (δ = 0.00 ppm) with the solvent resonance as the internal standard ($CDCl_3$: δ = 7.26 ppm). Data are reported as follows: chemical shift in ppm (δ), multiplicity (s = singlet, d = doublet, t = triplet, q = quartet, m = multiplet), coupling constant (Hz), and integration. Chemical shifts of ^{13}C NMR were reported in ppm with the solvent as the internal standard. High Resolution Mass measurement was performed on Waters Q-TOF 6520 mass spectrometer with electron spray ionization (ESI) as the ion source. Melting point (m.p.) was measured on a microscopic melting point apparatus.

2. Preparation of the Substrates

2.1 Involved substrates

1,3-dienes **1**

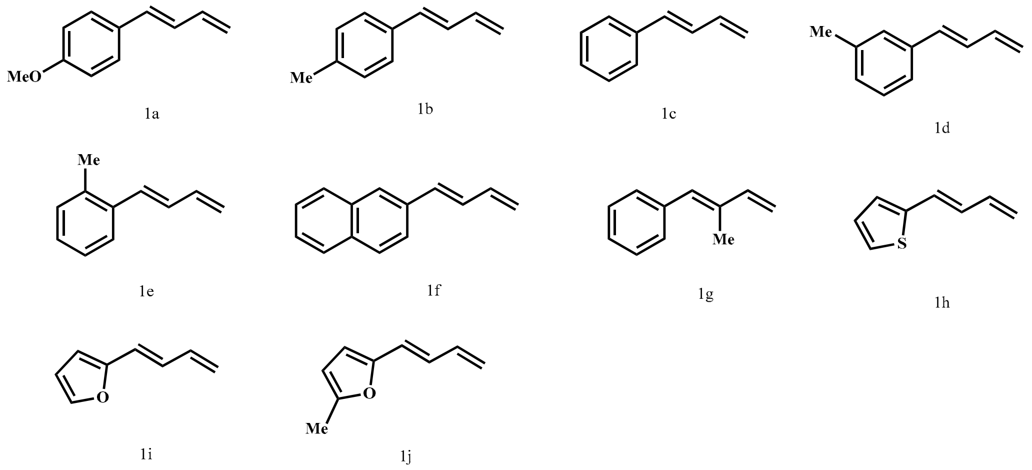

Figure S1. Structural formulas of substrate 1,3-dienes **1**

sulfinic acids **2**

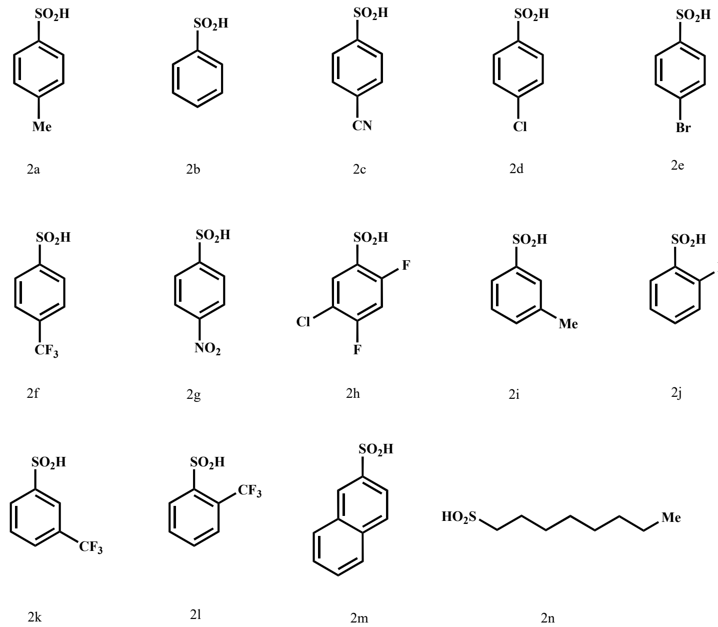

Figure S2. Structural formulas of substrate sulfinic acids **2**

2.2 General procedure for the synthesis of 1,3-dienes

1,3-dienes were prepared by the Wittig-Olefination reaction according to the previously reported literature[1] (general procedures A and B).

The following scheme shows procedure A:

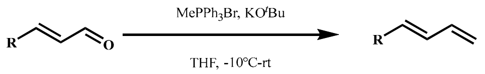

General Procedure A exemplified by the preparation of 1-(buta-1,3-dien-1-yl)4-methoxybenzene (**1a**): A stirred solution of Methyltriphenylphosphonium bromid e (15 mmol, 1.5 equiv) in dry THF (25 mL) was slowly added the KO^{t}Bu (1 5 mmol, 1.5 equiv) at room temperature (RT). The mixture was stirred at RT for 30 min until all phosphonium bromide had been dissolved. Afterwards, the mixture was transferred to -10 °C, (E)-3-(4-methoxyphenyl)acrylaldehyde (10 m mol, 1.0 equiv) was slowly added and stirred, and the mixture was allowed to warm up to RT for 12 hours. After the reaction was completed, the mixture w as diluted by adding PE (300 mL) and the solid phosphorus oxide precipitate was removed by filtration. Then the filtrate was concentrated *in vacuo* and the residue was purified by a silica gel to afford the desired 1,3-diene product.

The following scheme shows procedure B:

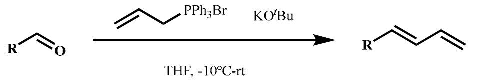

General Procedure B exemplified by the preparation of 1-(*m*-methylphenyl)-1,3-butadiene (**1d**): A stirred solution of Allyl triphenylphosphonium bromide (15 mmol, 1.5 equiv) in dry THF (25 mL) was slowly added the KO^{t}Bu (15 mmol, 1.5 equiv) at -10 °C. The mixture was stirred at -10 °C for 30 min until all phosphonium bromide had been dissolved. Afterwards, *m*-Tolualdehyde (10 mmol, 1.0 equiv) was slowly added and stirred, and the mixture was allowed to warm up to RT for 12 hours. After the reaction was completed, the mixture was diluted by adding PE (300 mL) and the solid phosphorus oxide precipitate was removed by filtration. Then the filtrate was concentrated *in vacuo* and the residue was purified by a silica gel to afford the desired 1,3-diene product.

2.3 General procedure for the synthesis of sulfinic acid 2

Sulfinic acids were prepared according to the previously reported literature.[2]

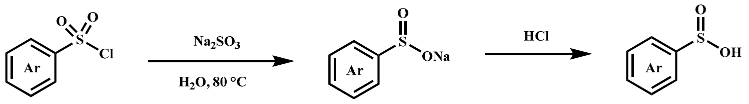

To a 50 mL flask containing Na_2SO_3 (30 mmol, 3 equiv) was added, and the solid was completely dissolved in pure water. Then substituted benzenesulfonyl chloride (10 mmol, 1.0 equiv) was added into reaction system. The mixture was stirred and kept at 80 °C for 12 h. Then, this aqueous solution was washed with chloroform twice, acidified with excess concentrated HCl solution, cooled and filtered. The white precipitate was recrystallized from water to give substituted benzenesulfinic acid.

3. Experimental procedure

3.1 General Procedure for the Hydrosulfonylation Reaction.

1,3-diene (0.20 mmol, 2 equiv), sulfinic acid (0.10 mmol, 1 equiv) were added to a 35 mL round bottom flask under air, followed by addition of DCM (3.0 mL). The formed mixture was stirred at room temperature for 8 h. Upon completion of the reaction, it was quenched with saturated ammonium chloride solution and the organic phase was washed with water and saturated sodium chloride solution sequentially. The solvent was then removed under reduced pressure. The resulting crude product was purified by flash chromatography on silica gel using petroleum ether and ethyl acetate as eluent in a ratio of PE/EA ranging from 10:1 to 4:1.

3.2 Procedure for the gram scale synthesis

1a(10mmol) + **2a**(5mmol) $\xrightarrow{\text{DCM 80ml, rt, 48h}}$ **3aa**, 1.12g (71%)

1,3-diene (10 mmol, 2 equiv), sulfinic acid (5 mmol, 1 equiv) were added to a 250 mL round bottomed flask under air, followed by addition of DCM (80 mL). The formed mixture was stirred and refluxed at room temperature for 48 h. When the reaction was finished, the solution was extracted with DCM (3×20 mL) and concentrated *in vacuo*. The pure product was obtained by flash chromatography on silica gel using petroleum ether and ethyl acetate as the eluent (PE/EA = 10:1 to 4:1).

3.3 Further transformation of the product to obtain tertiary sulfone[3]

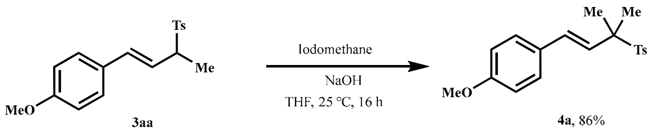

Typical procedure: To a stirred solution of **3aa** (0.1 mmol, 1.0 equiv) and NaOH (0.4 mmol, 4.0 equiv) in dry THF (1 mL) were slowly added the Iodomethane (0.12 mmol, 1.2 equiv.) at -10 °C. Then the mixture was allowed to warm up to RT for 16 hours. When the reaction was finished, the solution was diluted with DCM (3×5 mL), washed with water (30 mL), the combined organic layers were dried with anhydrous Na_2SO_4. The solvents were removed via rotary evaporator. The crude product was purified by flash column chromatography on silica gel (PE/EA = 4:1) to afford of product **4a**.

3.4 Epoxidation of allyl sulfone[4]

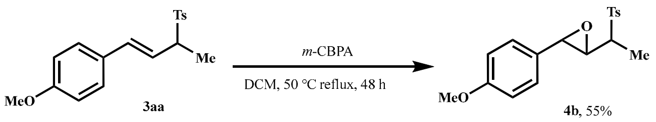

Procedure: To a 35 mL Schlenk tube were added **3aa** (0.2 mmol, 1.0 equiv), *m*-CBPA (0.4 mmol, 2.0 equiv) and DCM (2 mL). The resulting mixture was stirred under air at

50 °C for 48 h and monitored by TLC. And then quenched by addition of saturated NH_4Cl aqueous. The layers were separated and the aqueous phase was extracted with DCM (3×5 mL). The combined organic layer was dried over Na_2SO_4. Product **4b** was isolated by flash column chromatography on silica gel (PE/EA = 4:1).

Characterization of the generated allylic sulfones.

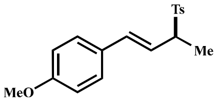

(*E*)-1-methoxy-4-(3-tosylbut-1-en-1-yl)benzene (3aa)
Yield 94%; White solid, m.p. 90-92 °C; ^{1}H NMR (600 MHz, $CDCl_3$) δ 7.71 (d, J = 8.3 Hz, 2H), 7.29 (d, J = 7.9 Hz, 2H), 7.22 (d, J = 8.3 Hz, 2H), 6.87 – 6.80 (m, 2H), 6.28 (d, J = 15.9 Hz, 1H), 5.92 (dd, J = 15.9, 8.3 Hz, 1H),3.78- 3.83 (m, 4H), 2.42 (s, 3H), 1.51 (d, J = 6.9 Hz, 3H).^{13}C NMR (150 MHz, $CDCl_3$) δ 159.7, 144.5, 135.7, 134.0, 129.4, 129.3, 128.7, 127.8, 119.8, 114.0, 64.1, 55.3, 21.6, 13.7. HRMS (ESI) calcd for $[C_{18}H_{20}O_3S+Na]^+$: 339.1031, Found: 339.1035.

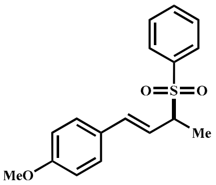

(*E*)-1-methoxy-4-(3-(phenylsulfonyl)but-1-en-1-yl)benzene (3ab)
Yield 90%; White solid, m.p. 81-82 °C; ^{1}H NMR (600 MHz, $CDCl_3$) δ 7.84 (d, J = 7.7 Hz, 2H), 7.61 (d, J = 7.0 Hz, 1H), 7.51 (t, J = 7.7 Hz, 2H), 7.22 (s, 2H), 6.84 (d, J = 7.0 Hz, 2H), 6.25 (d, J = 15.9 Hz, 1H), 5.92 (dd, J = 15.9, 8.2 Hz, 1H), 3.79-3.84 (m, 4H), 1.54 (d, J = 6.5 Hz, 3H).^{13}C NMR (150 MHz, $CDCl_3$) δ 159.7, 136.9, 136.0, 133.6, 130.8, 129.3, 128.8, 127.8, 119.5, 114.0, 64.2, 55.3, 13.6. HRMS (ESI) calcd for $[C_{17}H_{18}O_3S+Na]^+$: 325.0874, Found: 325.0859.

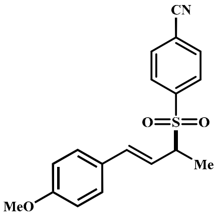

***(E)*-4-((4-(4-methoxyphenyl)but-3-en-2-yl)sulfonyl)benzonitrile (3ac)**
Yield 79%; Colorless oil; ^{1}H NMR (600 MHz, $CDCl_3$) δ 7.95 (d, J = 8.6 Hz, 2H), 7.80 (d, J = 8.6 Hz, 2H), 7.20 (d, J = 8.9 Hz, 2H), 6.85 (d, J = 8.9 Hz, 2H), 6.22 (d, J = 15.9 Hz, 1H), 5.89 (dd, J = 15.9, 8.6 Hz, 1H), 3.81-3.87 (m, 4H), 1.57 (d, J = 6.9 Hz, 3H).^{13}C NMR (150 MHz, $CDCl_3$) δ 160.1, 141.4, 136.8, 132.5, 130.1, 128.1, 127.9, 118.7, 117.4, 117.2, 114.2, 64.5, 55.3, 13.3. HRMS (ESI) calcd for $[C_{18}H_{17}NO_3S+Na]^+$: 350.0827, Found: 350.0828.

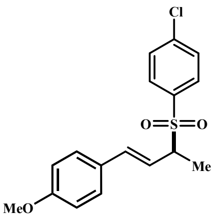

(*E*)-1-chloro-4-((4-(4-methoxyphenyl)but-3-en-2-yl)sulfonyl)benzene (3ad)
Yield 76%; White solid, m.p. 76-77 °C; ^{1}H NMR (600 MHz, $CDCl_3$) δ 7.76 (d, J = 8.8 Hz, 2H), 7.48 (d, J = 8.8 Hz, 2H), 7.22 (d, J = 8.8 Hz, 2H), 6.85 (d, J = 8.8 Hz, 2H), 6.26 (d, J = 15.9 Hz, 1H), 5.91 (dd, J = 15.9, 8.3 Hz, 1H), 3.80-3.82 (m, 4H), 1.54 (d, J = 6.9 Hz, 3H). ^{13}C NMR (150 MHz, $CDCl_3$) δ 159.9, 140.4, 136.3, 135.5, 130.8, 129.1, 128.4, 127.9, 119.3, 114.1, 64.3, 55.3, 13.6. HRMS (ESI) calcd for $[C_{17}H_{17}ClO_3S+Na]^+$: 359.0485, Found: 359.0482.

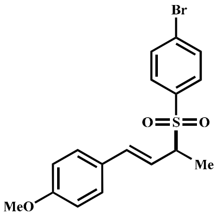

(*E*)-1-bromo-4-((4-(4-methoxyphenyl)but-3-en-2-yl)sulfonyl)benzene (3ae)
Yield 94%; White solid, m.p. 87-88 °C; ^{1}H NMR (600 MHz, $CDCl_3$) δ 7.78 – 7.57 (m, 4H), 7.22 (d, J = 8.8 Hz, 2H), 6.84 (d, J = 8.8 Hz, 2H), 6.27 (d, J = 15.9 Hz, 1H), 5.90 (dd, J = 15.9, 8.3 Hz, 1H), 3.80-3.83 (m, 4H), 1.53 (d, J = 7.0 Hz, 3H).^{13}C NMR (150 MHz, $CDCl_3$) δ 159.9, 136.3, 132.1, 131.8, 130.9, 129.0, 128.4, 127.9, 119.2, 114.1, 64.3, 55.3, 13.5. HRMS (ESI) calcd for $[C_{17}H_{17}BrO_3S+Na]^+$: 402.9979, Found: 402.9970.

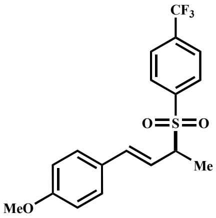

(*E*)-1-methoxy-4-(3-((4-(trifluoromethyl)phenyl)sulfonyl)but-1-en-1-yl)benzene (3af)
Yield 62%; White solid, m.p. 101-102 °C; ^{1}H NMR (600 MHz, $CDCl_3$) δ 7.97 (d, J = 8.1 Hz, 2H), 7.77 (d, J = 8.1 Hz, 2H), 7.21 (d, J = 8.7 Hz, 2H), 6.84 (d, J = 8.7 Hz, 2H), 6.25 (d, J = 15.9 Hz, 1H), 5.91 (dd, J = 15.9, 8.4 Hz, 1H), 3.89 – 3.83 (m, 1H), 3.81 (s, 3H), 1.56 (d, J = 7.0 Hz, 3H).^{13}C NMR (150 MHz, $CDCl_3$) δ 160.1, 140.7, 136.6, 135.4, 130.0, 128.3, 127.9, 125.9, 125.9, 118.9, 114.2, 64.4, 55.3, 13.5. HRMS (ESI) calcd for $[C_{18}H_{17}F_3O_3S+Na]^+$: 393.0748, Found: 393.0753.

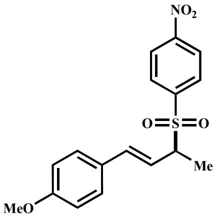

(*E*)-1-methoxy-4-(3-((4-nitrophenyl)sulfonyl)but-1-en-1-yl)benzene (3ag)

Yield 81%; yellow solid m.p. 151-153 °C; ^{1}H NMR (600 MHz, CDCl3) δ 8.34 (d, J = 9.0 Hz, 2H), 8.03 (d, J = 9.0 Hz, 2H), 7.20 (d, J = 8.8 Hz, 2H), 6.84 (d, J = 8.8 Hz, 2H), 6.25 (d, J = 15.9 Hz, 1H), 5.91 (dd, J = 15.9, 8.4 Hz, 1H), 3.91 – 3.86 (m, 1H), 3.81 (s, 3H), 1.58 (d, J = 7.0 Hz, 3H).^{13}C NMR (150 MHz, CDCl3) δ 160.2, 150.8, 142.9, 136.9, 130.8, 128.0, 127.9, 123.9, 118.6, 114.2, 64.6, 55.3, 13.4. HRMS (ESI) calcd for $[C_{17}H_{17}NO_5S+Na]^+$: 370.0725, Found: 370.0688.

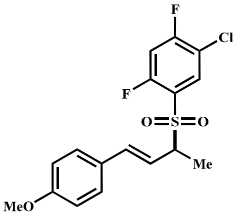

(*E*)-1-chloro-2,4-difluoro-5-((4-(4-methoxyphenyl)but-3-en-2-yl)sulfonyl)be nzene (3ah)

Yield 79%; White solid, m.p. 64-66 °C; ^{1}H NMR (600 MHz, CDCl3) δ 7.98 – 7.93 (t, J = 8.6 Hz, 1H), 7.22 (d, J = 8.9 Hz, 2H), 7.04 (t, J = 8.6 Hz, 1H), 6.83 (d, J = 8.9 Hz, 2H), 6.39 (d, J = 15.8 Hz, 1H), 5.96 – 5.90 (m, 1H), 4.11 – 4.03 (m, 1H), 3.80 (s, 3H), 1.61 (d, J = 6.9 Hz, 3H).^{13}C NMR (150 MHz, CDCl3) δ 162.5, 160.1, 157.6, 136.9, 133.1, 128.2, 128.0, 118.6, 114.1, 106.9, 106.7, 106.5, 64.7, 55.3, 13.2. HRMS (ESI) calcd for $[C_{17}H_{15}ClF_2O_3S+Na]^+$: 395.0296, Found: 395.0278.

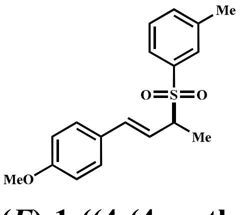

(*E*)-1-((4-(4-methoxyphenyl)but-3-en-2-yl)sulfonyl)-3-methylbenzene (3ai)

Yield 74%; Colorless oil; ^{1}H NMR (600 MHz, CDCl3) δ 7.63 (d, J = 8.9 Hz, 2H), 7.40 (s, 2H), 7.21 (d, J = 8.9 Hz, 2H), 6.83 (d, J = 8.9 Hz, 2H), 6.26 (d, J = 15.9 Hz, 1H), 5.91 (dd, J = 15.9, 8.3 Hz, 1H), 3.79-3.83 (m, 4H), 2.36 (s, 3H), 1.52 (d, J = 6.9 Hz, 3H).^{13}C NMR (150 MHz, CDCl3) δ 159.8, 139.0, 136.8, 135.9, 134.4, 129.7, 128.7, 128.6, 127.8, 126.4, 119.7, 114.0, 64.2, 55.3, 21.2, 13.6. HRMS (ESI) calcd for $[C_{18}H_{20}O_3S+Na]^+$: 339.1031, Found: 339.1030.

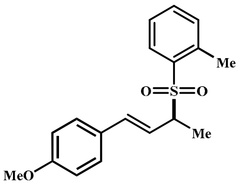

(*E*)-1-((4-(4-methoxyphenyl)but-3-en-2-yl)sulfonyl)-2-methylbenzene (3aj)

Yield 83%; Colorless oil; ^{1}H NMR (600 MHz, $CDCl_3$) δ 7.90 (d, *J* = 6.3 Hz, 1H), 7.46 (t, *J* = 6.3 Hz, 1H), 7.31 – 7.27 (m, 2H), 7.17 (d, *J* = 8.7 Hz, 2H), 6.81 (d, *J* = 8.7 Hz, 2H), 6.21 (d, *J* = 15.9 Hz, 1H), 5.91 (dd, *J* = 15.9, 8.7 Hz, 1H), 3.90 – 3.85 (m, 1H), 3.79 (s, 3H), 2.68 (s, 3H), 1.55 (d, *J* = 7.0 Hz, 3H).^{13}C NMR (150 MHz, $CDCl_3$) δ 159.8, 138.7, 136.0, 133.5, 132.6, 131.5, 129.4, 128.6, 127.9, 126.3, 119.7, 114.0, 63.8, 55.3, 20.8, 13.3. HRMS (ESI) calcd for $[C_{18}H_{20}O_3S+Na]^+$: 339.1031, Found: 339.1031.

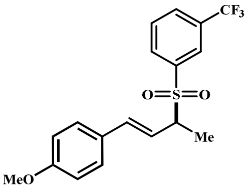

***(E)*-1-((4-(4-methoxyphenyl)but-3-en-2-yl)sulfonyl)-3-(trifluoromethyl)benzene (3ak)**

Yield 67%; White solid, m.p. 103-104 °C; ^{1}H NMR (600 MHz, $CDCl_3$) δ 8.12 (s, 1H) 8.02 (d, J = 7.9 Hz, 1H), 7.87 (d, J = 7.9 Hz, 1H)7.65 (t, J = 7.9 Hz, 1H), 7.20 (d, J = 8.7 Hz, 2H), 6.83 (d, J = 8.7 Hz, 2H), 6.24 (d, J = 15.9 Hz, 1H), 5.90 (dd, J = 15.9, 8.5 Hz, 1H),3.83 – 3.88 (m, 1H) 3.81 (s, 3H), 1.56 (d, J = 6.9 Hz, 3H).^{13}C NMR (150 MHz, $CDCl_3$) δ 160.0, 138.3, 136.7, 132.7, 131.7, 130.3, 129.6, 128.2, 127.9, 126.5, 126.5, 119.0, 114.1, 64.5, 55.3, 13.4. HRMS (ESI) calcd for $[C_{18}H_{17}F_3O_3S+Na]^+$: 393.0748, Found: 393.0745.

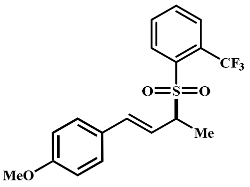

***(E)*-1-((4-(4-methoxyphenyl)but-3-en-2-yl)sulfonyl)-2-(trifluoromethyl)benzene (3al)**

Yield 64%; White solid, m.p. 100-101 °C; ^{1}H NMR (600 MHz, $CDCl_3$) δ 8.12 (d, J = 9.4 Hz, 1H), 7.91 (d, J = 7.7 Hz, 1H), 7.73 – 7.67 (m, 1H), 7.64 (t, J = 7.7 Hz, 1H), 7.17 (d, J = 8.7 Hz, 2H), 6.81 (d, J = 8.9 Hz, 2H), 6.19 (d, J = 15.8 Hz, 1H), 5.90 (dd, J = 15.8, 9.0 Hz, 1H), 4.16 – 4.08 (m, 1H), 3.79 (s, 3H), 1.60 (d, J = 6.9 Hz, 3H).^{13}C NMR (150 MHz, $CDCl_3$) δ 159.9, 136.6, 134.4, 133.5, 131.9, 129.4, 128.4, 128.3, 127.9, 123.6, 121.8, 119.6, 114.0, 64.6, 55.3, 13.4. HRMS (ESI) calcd for $[C_{18}H_{17}F_3O_3S +Na]^+$: 393.0748, Found: 393.0751.

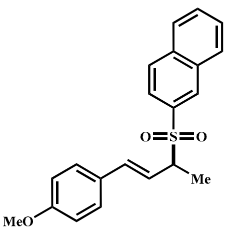

(*E*)-2-((4-(4-methoxyphenyl)but-3-en-2-yl)sulfonyl)naphthalene (3am)

Yield 81%; White solid, m.p. 119-120.5 °C; ^{1}H NMR (600 MHz, $CDCl_3$) δ 8.43 (s, 1H), 7.92 (dd, J = 14.5, 8.7 Hz, 3H), 7.81 (dd, J = 8.7, 1.8 Hz, 1H), 7.69 – 7.63 (m, 1H), 7.60 (td, J = 7.6, 1.8 Hz, 1H), 7.18 (d, J = 8.7 Hz, 2H), 6.81 (d, J = 8.7 Hz, 2H), 6.28 (d, J = 15.9 Hz, 1H), 5.97 (dd, J = 15.9, 8.3 Hz, 1H), 3.89-3.94 (m, 1H), 3.79 (s, 3H), 1.56 (d, J = 6.9 Hz, 3H). ^{13}C NMR (150 MHz, $CDCl_3$) δ 159.8, 136.0, 135.3, 134.1, 132.0, 131.2, 129.4, 129.2, 128.8, 128.6, 127.9, 127.9, 127.5, 124.1, 119.6, 114.0, 64.3, 55.3, 13.8. HRMS (ESI) calcd for $[C_{21}H_{20}O_3S+Na]^+$: 375.1031, Found: 375.1034.

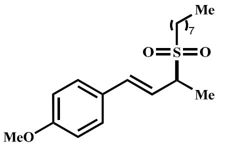

(*E*)-1-methoxy-4-(3-(octylsulfonyl)but-1-en-1-yl)benzene (3an)

Yield 68%; Colorless oil; ^{1}H NMR (600 MHz, $CDCl_3$) δ 7.35 (d, J = 8.7 Hz, 2H), 6.88 (d, J = 8.9 Hz, 2H), 6.60 (d, J = 15.8 Hz, 1H), 6.07 (dd, J = 15.9, 9.0 Hz, 1H), 3.82 (s, 3H), 3.74-3.78 (m, 1H), 2.99 – 2.90 (m, 2H), 1.88 – 1.78 (m, 2H), 1.58 (d, J = 6.9 Hz, 3H), 1.26 (q, J = 6.3 Hz, 10H), 0.86 (t, J = 7.2 Hz, 3H). ^{13}C NMR (150 MHz, CDCl3) δ 160.0, 135.4, 128.3, 128.0, 120.7, 114.2, 61.7, 55.4, 49.6, 31.7, 29.0, 28.9, 28.6, 22.6, 21.4, 14.1, 12.6. HRMS (ESI) calcd for $[C_{19}H_{30}O_3S+Na]^+$: 361.1813, Found: 361.1825.

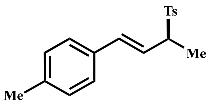

(*E*)-1-methyl-4-((4-(p-tolyl)but-3-en-2-yl)sulfonyl)benzene (3ba)

Yield 54%; White solid, m.p. 95-96 °C; ^{1}H NMR (600 MHz, $CDCl_3$) δ 7.71 (d, J = 7.9 Hz, 2H), 7.29 (d, J = 7.9 Hz, 2H), 7.18 (d, J = 7.9 Hz, 2H), 7.11 (d, J = 7.9 Hz, 2H), 6.29 (d, J = 15.9 Hz, 1H), 6.02 (dd, J = 15.9, 8.2 Hz, 1H), 3.79-3.84 (m, 1H), 2.42 (s, 3H), 2.33 (s, 3H), 1.52 (d, J = 7.0 Hz, 3H). ^{13}C NMR (150 MHz, $CDCl_3$) δ 144.5, 138.3, 136.2, 134.0, 133.2, 129.4, 129.3, 126.5, 121.2, 64.1, 21.6, 21.2, 13.6. HRMS (ESI) calcd for $[C_{18}H_{20}O_2S+Na]^+$: 323.1082, Found: 323.1089.

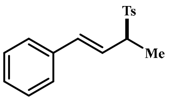

(*E*)-1-methyl-4-((4-phenylbut-3-en-2-yl)sulfonyl)benzene (3ca)

Yield 10%; White solid, m.p. 90-91 °C; ^{1}H NMR (600 MHz, $CDCl_3$) δ 7.71 (d, J = 8.2 Hz, 2H), 7.33 – 7.26 (m, 7H), 6.34 (d, J = 15.9 Hz, 1H), 6.08 (dd, J = 15.9, 8.2 Hz, 1H),

3.81-3.86 (m, 1H), 2.43 (s, 3H), 1.53 (d, *J* = 6.9 Hz, 3H). ^{13}C NMR (150 MHz, $CDCl_3$) *δ* 159.8, 136.9, 136.0, 133.6, 130.8, 129.3, 128.8, 127.9, 119.6, 114.0, 64.2, 55.3, 13.6. HRMS (ESI) calcd for $[C_{17}H_{18}O_2S+Na]^+$ 309.0925, Found: 309.0923.

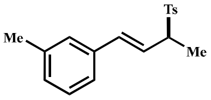

(*E*)-1-methyl-3-(3-tosylbut-1-en-1-yl)benzene (3da)
Yield 24%; Colorless oil; ^{1}H NMR (600 MHz, $CDCl_3$) *δ* 7.71 (d, *J* = 8.2 Hz, 2H), 7.30 (d, *J* = 8.2 Hz, 2H), 7.20 (t, *J* = 7.6 Hz, 1H), 7.12 (s, 1H), 7.08 (t, *J* = 7.6 Hz, 2H), 6.29 (d, *J* = 15.9 Hz, 1H), 6.07 (dd, *J* = 15.9, 8.2 Hz, 1H), 3.80-3.85 (m, 1H), 2.42 (s, 3H), 2.33 (s, 3H), 1.53 (d, *J* = 7.2 Hz, 3H).^{13}C NMR (150 MHz, $CDCl_3$) *δ* 144.6, 138.2, 136.4, 135.9, 134.0, 129.5, 129.3, 129.1, 128.5, 127.2, 123.8, 122.0, 64.1, 21.6, 21.4, 13.6. HRMS (ESI) calcd for $[C_{18}H_{20}O_2S+Na]^+$: 323.1082, Found: 323.1089.

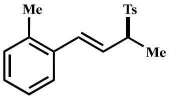

(*E*)-1-methyl-2-(3-tosylbut-1-en-1-yl)benzene (3ea)
Yield 27%; Colorless oil; ^{1}H NMR (600 MHz, $CDCl_3$) *δ* 7.73 (d, *J* = 8.0 Hz, 2H), 7.37 – 7.32 (m, 1H), 7.31 (d, *J* = 8.0 Hz, 2H), 7.17 (d, *J* = 3.7 Hz, 2H), 7.13 – 7.08 (m, 1H), 6.52 (d, *J* = 15.8 Hz, 1H), 5.94 (dd, *J* = 15.8, 8.5 Hz, 1H), 3.83-3.89 (m, 1H), 2.42 (s, 3H), 2.14 (s, 3H), 1.56 (s, 3H).^{13}C NMR (150 MHz, $CDCl_3$) *δ* 144.6, 135.6, 135.0, 134.4, 134.1, 130.3, 129.4, 129.3, 128.2, 126.2, 125.8, 123.8, 64.4, 21.6, 19.4, 13.6. HRMS (ESI) calcd for $[C_{18}H_{20}O_2S+Na]^+$: 323.1082, Found: 323.1083.

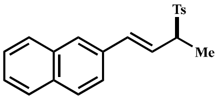

(*E*)-2-(3-tosylbut-1-en-1-yl)naphthalene (3fa)
Yield 67%; White solid, m.p. 122-124 °C; ^{1}H NMR (600 MHz, $CDCl_3$) *δ* 7.81 – 7.76 (m, 3H), 7.73 (d, *J* = 8.2 Hz, 2H), 7.63 (s, 1H), 7.52 – 7.49 (m, 1H), 7.46 (m, 2H), 7.28 (d, *J* = 8.2 Hz, 2H), 6.48 (d, *J* = 15.9 Hz, 1H), 6.21 (dd, *J* = 15.9, 8.2 Hz, 1H), 3.86-3.91 (m, 1H), 2.41 (s, 3H), 1.57 (d, *J* = 7.2 Hz, 3H).^{13}C NMR (150 MHz, $CDCl_3$) *δ* 144.6, 136.3, 134.0, 133.4, 133.3, 133.2, 129.5, 129.3, 128.4, 128.1, 127.7, 126.9, 126.4, 126.3, 123.3, 122.6, 64.2, 21.6, 13.6. HRMS (ESI) calcd for $[C_{21}H_{20}O_2S+Na]^+$: 359.1082, Found: 359.1097.

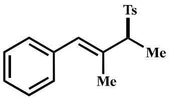

(*E*)-1-methyl-4-((3-methyl-4-phenylbut-3-en-2-yl)sulfonyl)benzene (3ga)
Yield 36%; Colorless oil; ^{1}H NMR (600 MHz, $CDCl_3$) *δ* 7.74 (d, *J* = 8.1 Hz, 2H), 7.30 (dd, *J* = 16.9, 8.1 Hz, 4H), 7.21 (t, *J* = 7.5 Hz, 1H), 7.02 (d, *J* = 7.5 Hz, 2H), 6.10 (s,

1H), 3.80 (q, J = 7.1 Hz, 1H), 2.44 (s, 3H), 1.90 (d, J = 1.5 Hz, 3H), 1.62 (d, J = 7.1 Hz, 3H).^{13}C NMR (150 MHz, $CDCl_3$) δ 144.5, 136.7, 134.3, 133.2, 131.4, 129.4, 129.2, 128.7, 128.1, 126.9, 69.3, 21.6, 15.8, 12.4. HRMS (ESI) calcd for[$C_{18}H_{20}O_2S$+Na]$^+$: 323.1082, Found: 323.1090.

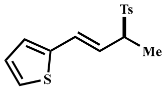

(*E*)-2-(3-tosylbut-1-en-1-yl)thiophene (3ha)

Yield 72%; White solid, m.p. 84-85 °C; ^{1}H NMR (600 MHz, $CDCl_3$) δ 7.71 (d, J = 8.0 Hz, 2H), 7.31 (d, J = 8.0 Hz, 2H), 7.19 (s, 1H), 6.95 (dd, J = 5.1, 3.4 Hz, 1H), 6.91 (d, J = 3.4 Hz, 1H), 6.48 (d, J = 15.5 Hz, 1H), 5.88 (dd, J = 15.5, 8.2 Hz, 1H), 3.76-3.82 (m, 1H), 2.43 (s, 3H), 1.50 (d, J = 7.2 Hz, 3H).^{13}C NMR (150 MHz, $CDCl_3$) δ 144.7, 140.9, 133.9, 129.5, 129.4, 129.3, 127.5, 126.8, 125.3, 121.5, 63.9, 21.6, 13.6. HRMS (ESI) calcd for [$C_{15}H_{16}O_2S_2$+Na]$^+$: 315.0489, Found: 315.0492.

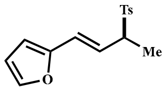

(*E*)-2-(3-tosylbut-1-en-1-yl)furan (3ia)

Yield 67%; Colorless oil; ^{1}H NMR (600 MHz, $CDCl_3$) δ 7.72 (d, J = 8.3 Hz, 2H), 7.35 (d, J = 1.3 Hz, 1H), 7.31 (d, J = 8.3 Hz, 2H), 6.36 (dd, J = 3.3, 1.3 Hz, 1H), 6.26 – 6.17 (m, 2H), 6.01 (dd, J = 15.8, 8.3 Hz, 1H), 3.77-3.83 (m, 1H), 2.43 (s, 3H), 1.49 (d, J = 6.9 Hz, 3H).^{13}C NMR (150 MHz, $CDCl_3$) δ 151.6, 144.7, 142.7, 133.9, 129.5, 129.4, 124.2, 120.4, 111.4, 109.4, 63.9, 21.6, 13.7. HRMS (ESI) calcd for [$C_{15}H_{16}O_3S$+Na]$^+$: 299.0718, Found: 299.0721.

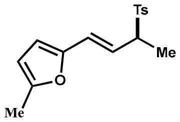

(E)-2-methyl-5-(3-tosylbut-1-en-1-yl)furan (3ja)

Yield 61%; White solid, m.p. 95-96 °C ^{1}H NMR (600 MHz, $CDCl_3$) δ 7.72 (d, J = 8.3 Hz, 2H), 7.31 (d, J = 8.3 Hz, 2H), 6.13 – 6.06 (m, 2H), 5.99 – 5.91 (m, 2H), 3.72-3.80 (m, 1H), 2.43 (s, 3H), 2.29 (s, 3H), 1.47 (d, J = 7.0 Hz, 3H).^{13}C NMR (150 MHz, $CDCl_3$) δ 152.9, 150.1, 144.6, 134.1, 129.5, 129.4, 124.3, 118.5, 110.7, 107.6, 64.0, 21.7, 13.8, 13.7. HRMS (ESI) calcd for[$C_{16}H_{18}O_3S$+Na]$^+$: 313.0874, Found: 313.0873.

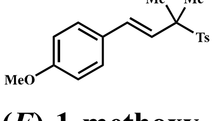

(*E*)-1-methoxy-4-(3-methyl-3-tosylbut-1-en-1-yl)benzene (4a)

Yield 86%; White solid, m.p. 109-110 °C; ^{1}H NMR (600 MHz, CDCl3) δ 7.67 (d, J = 8.8 Hz, 2H), 7.29 – 7.24 (m, 4H), 6.86 (d, J = 8.8 Hz, 2H), 6.26 (d, J = 16.3 Hz, 1H), 6.13 (d, J = 16.3 Hz, 1H), 3.82 (s, 3H), 2.41 (s, 3H), 1.53 (s, 6H).^{13}C NMR (150 MHz,

$CDCl_3$) δ 159.7, 144.5, 132.8, 132.4, 130.6, 129.5, 129.1, 128.9, 127.9, 127.0, 125.6, 114.1, 64.6, 55.3, 29.7, 21.7, 21.3. HRMS (ESI) calcd for [$C_{19}H_{22}O_3S+Na]^+$: 353.1187, Found: 353.1186.

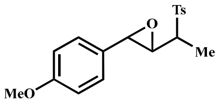

(*E*)-2-(4-methoxyphenyl)-3-(1-tosylethyl)oxirane (4b)

Yield 55%; White solid, m.p. 137-138 °C; ^1H NMR (600 MHz, $CDCl_3$) δ 7.54 (d, J = 8.3 Hz, 2H), 7.27 (d, J = 8.3 Hz, 2H), 7.18 (d, J = 8.9 Hz, 2H), 6.83 (d, J = 8.9 Hz, 2H), 3.83 – 3.79 (m, 4H), 3.39 (dd, J = 7.5, 2.0 Hz, 1H), 2.75 (qd, J = 5.2, 2.0 Hz, 1H), 2.43 (s, 3H), 1.29 (d, J = 5.2 Hz, 3H). ^{13}C NMR (150 MHz, $CDCl_3$) δ 160.3, 145.0, 134.8, 131.3, 129.6, 129.1, 121.7, 114.1, 72.5, 56.3, 55.4, 55.3, 21.7, 17.1. HRMS (ESI) calcd for [$C_{18}H_{20}O_4S+Na]^+$: 355.0980, Found: 355.0986.

4. ^{1}H and ^{13}C-NMR Spectra of the Title Compounds

3aa

^{1}H NMR

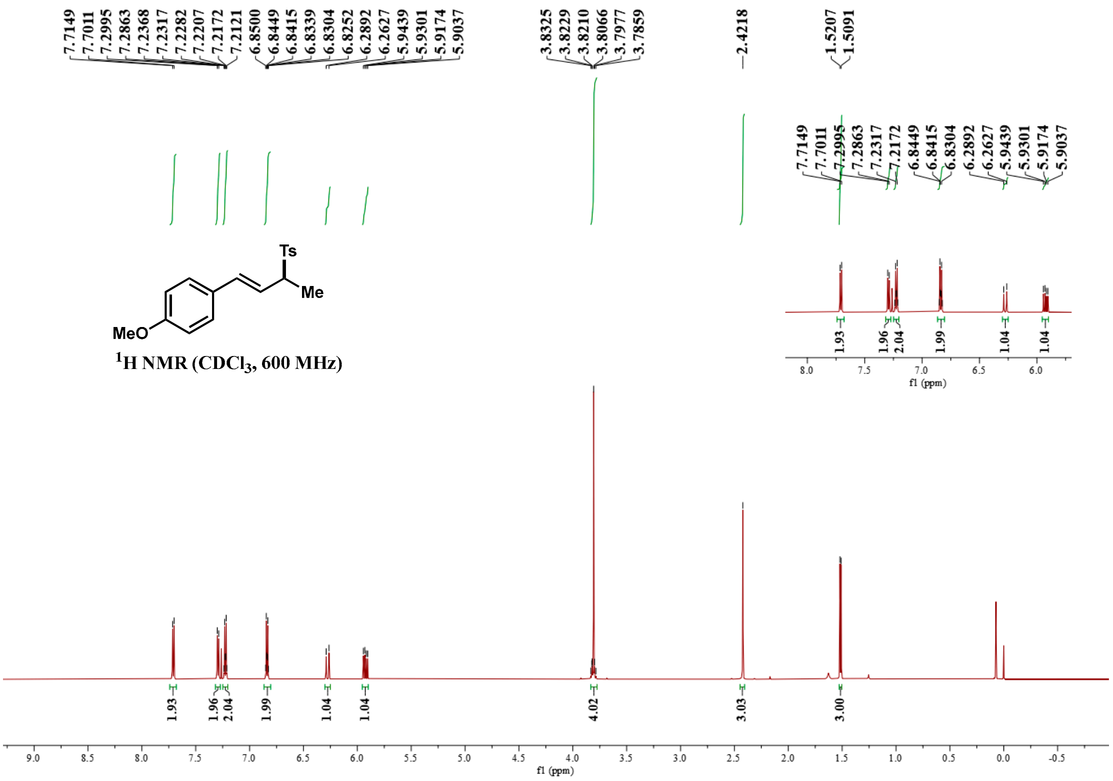

^{13}C NMR

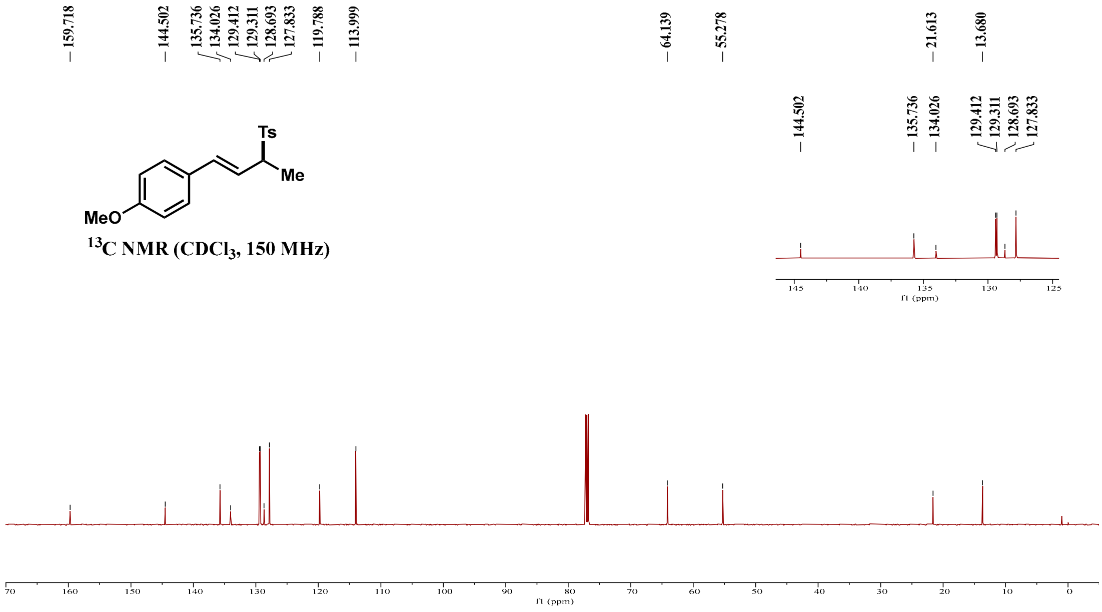

Figure S3. ^{1}H NMR and ^{13}C NMR spectra of **3aa** in $CDCl_3$

3ab

^{1}H NMR

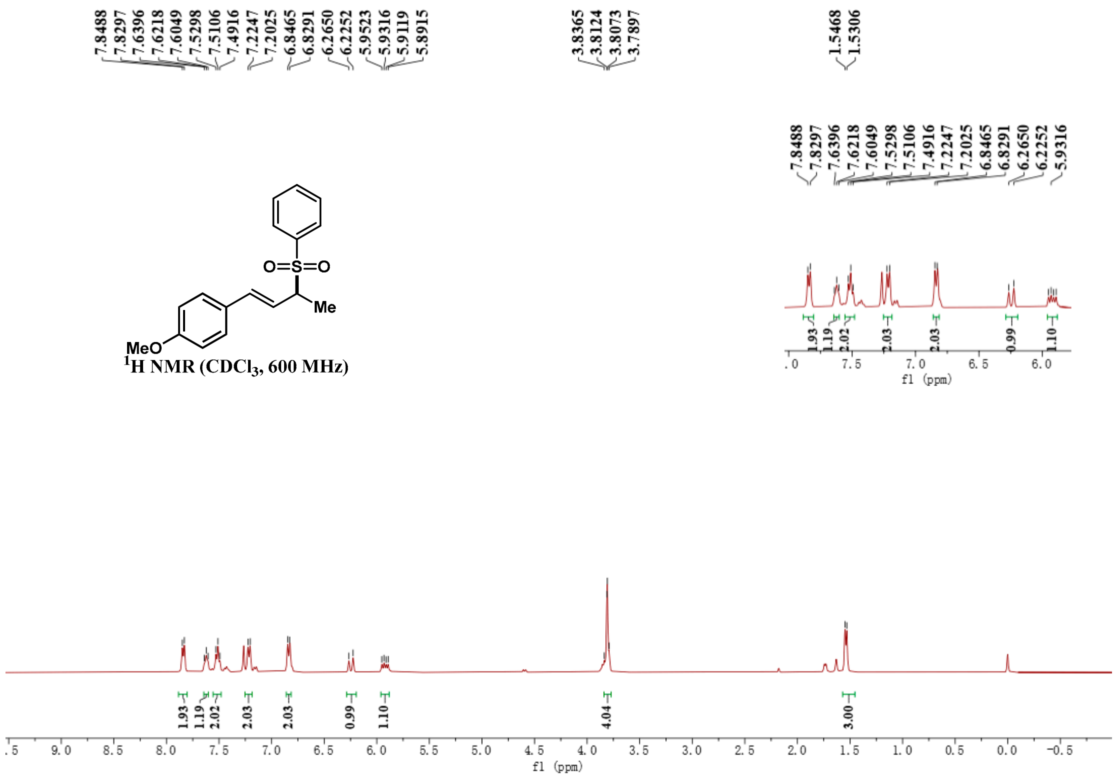

^{13}C NMR

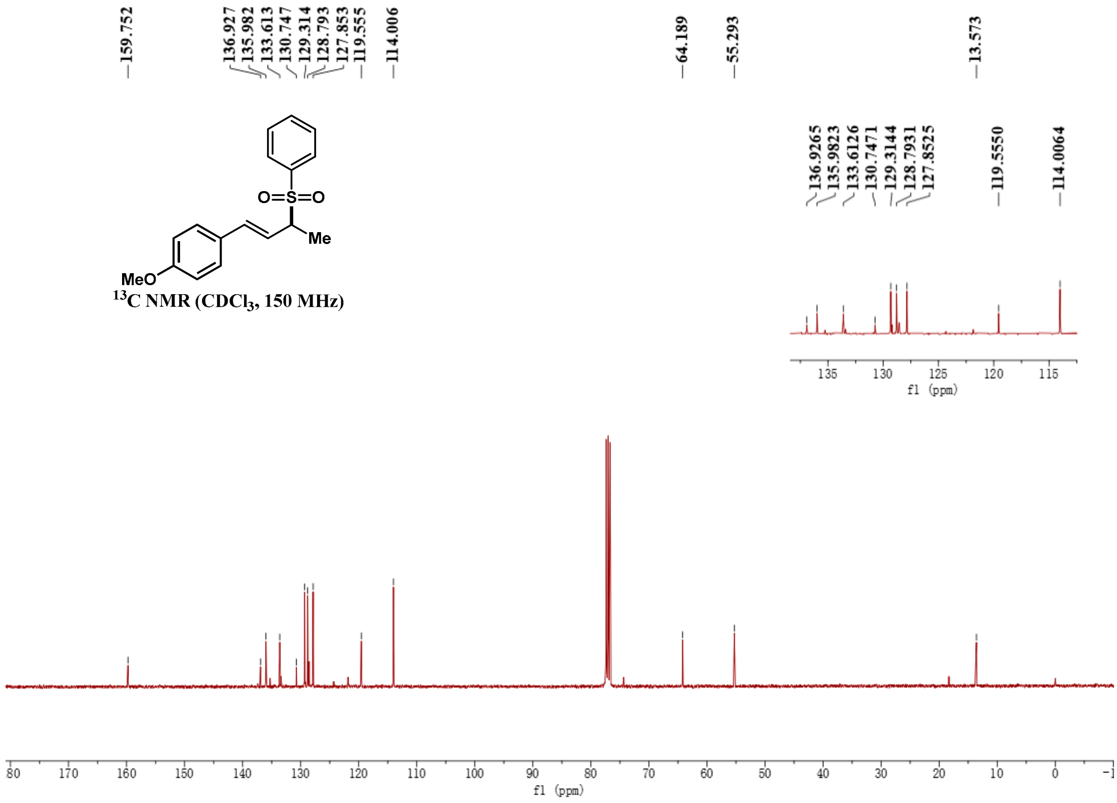

Figure S4. ^{1}H NMR and ^{13}C NMR spectra of **3ab** in CDCl$_3$

3ac

^{1}H NMR

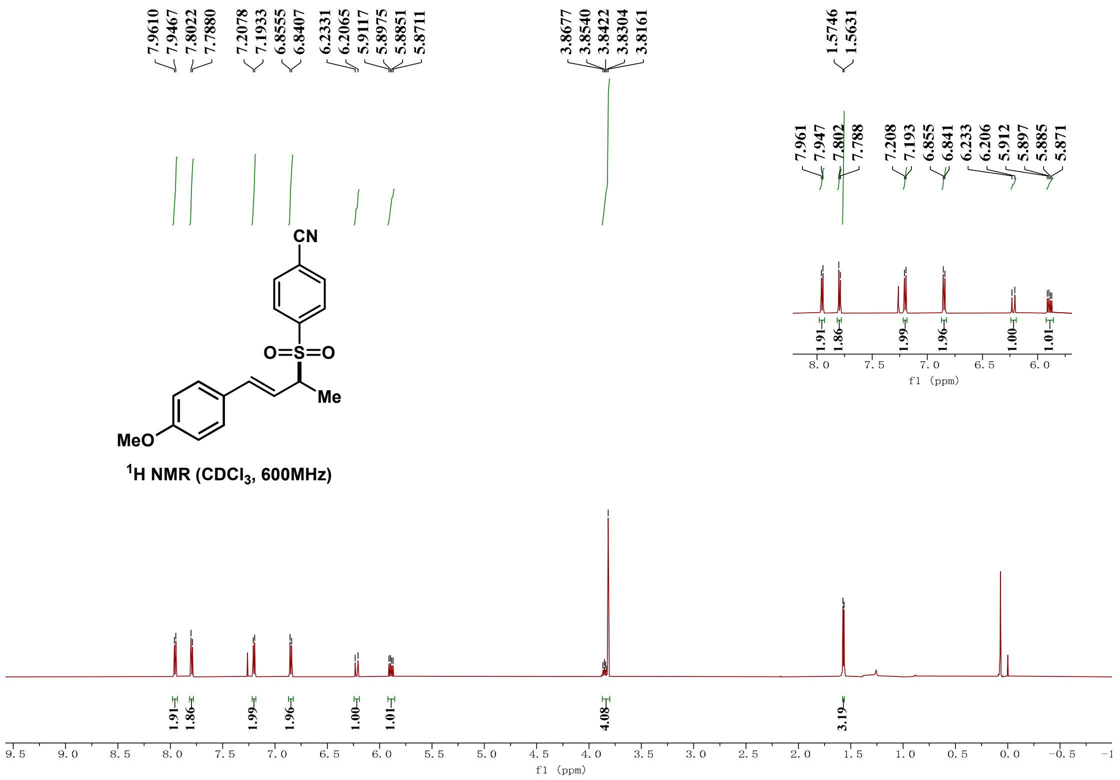

^{13}C NMR

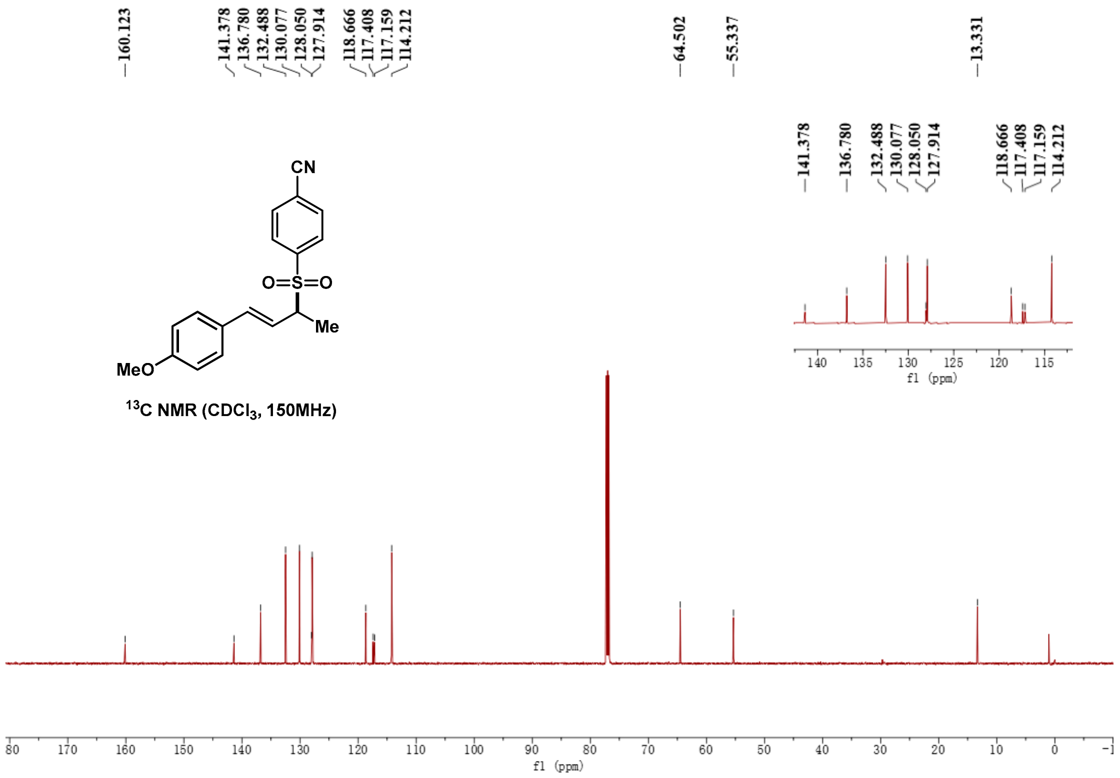

Figure S5. ^{1}H NMR and ^{13}C NMR spectra of **3ac** in $CDCl_3$

3ad

^{1}H NMR

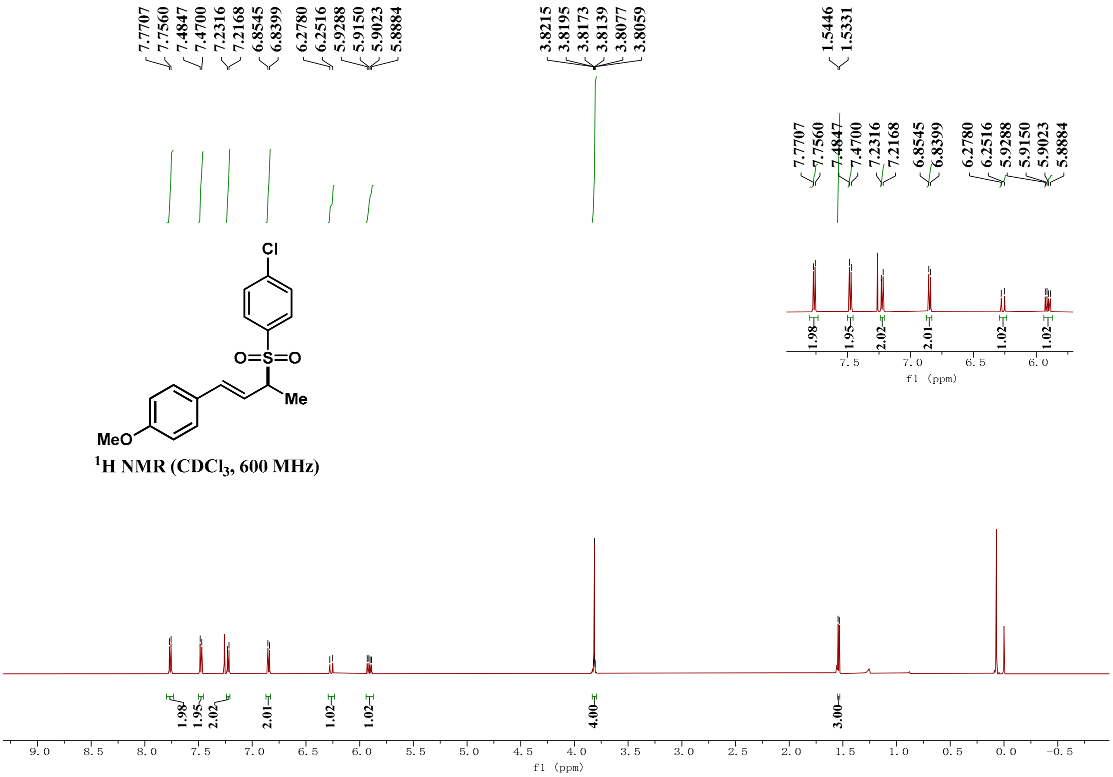

^{13}C NMR

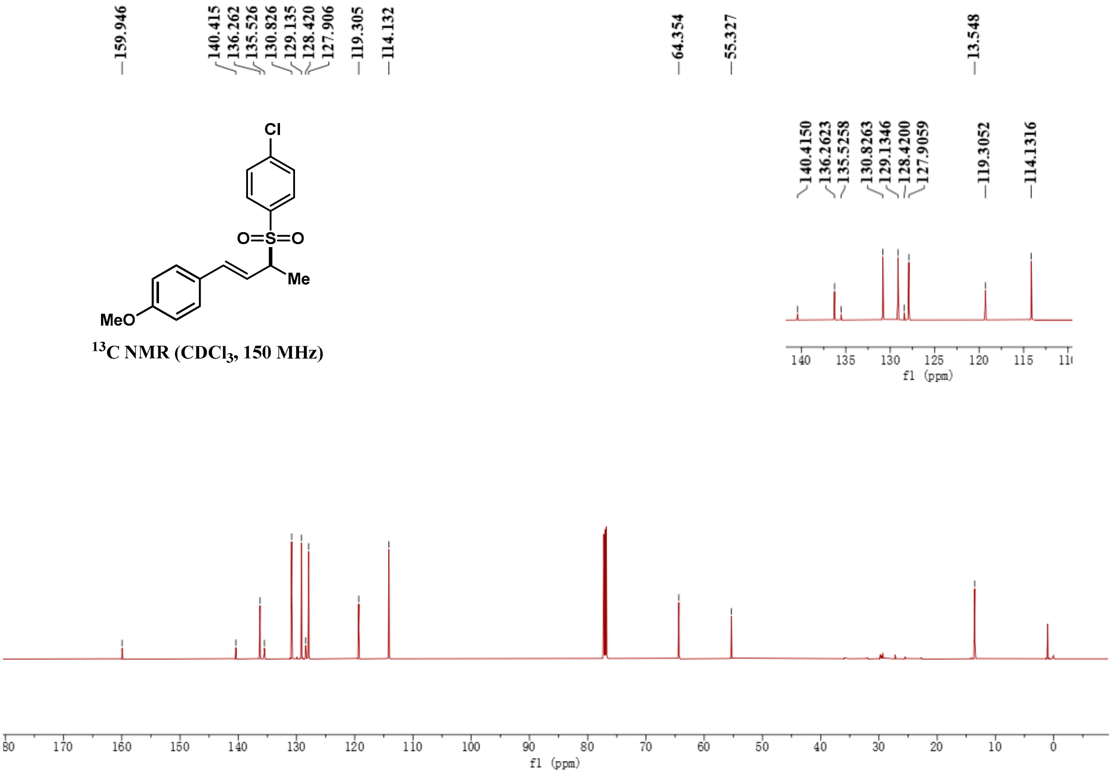

Figure S6. ^{1}H NMR and ^{13}C NMR spectra of **3ad** in $CDCl_3$

3ae

^{1}H NMR

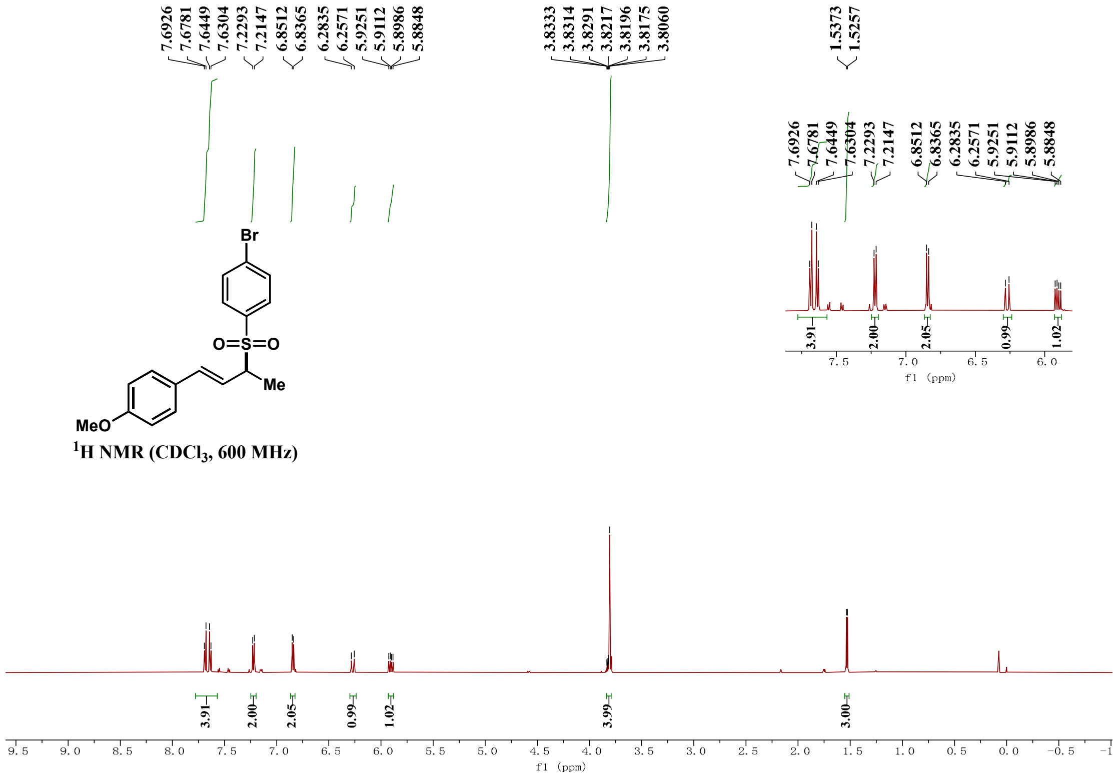

^{13}C NMR

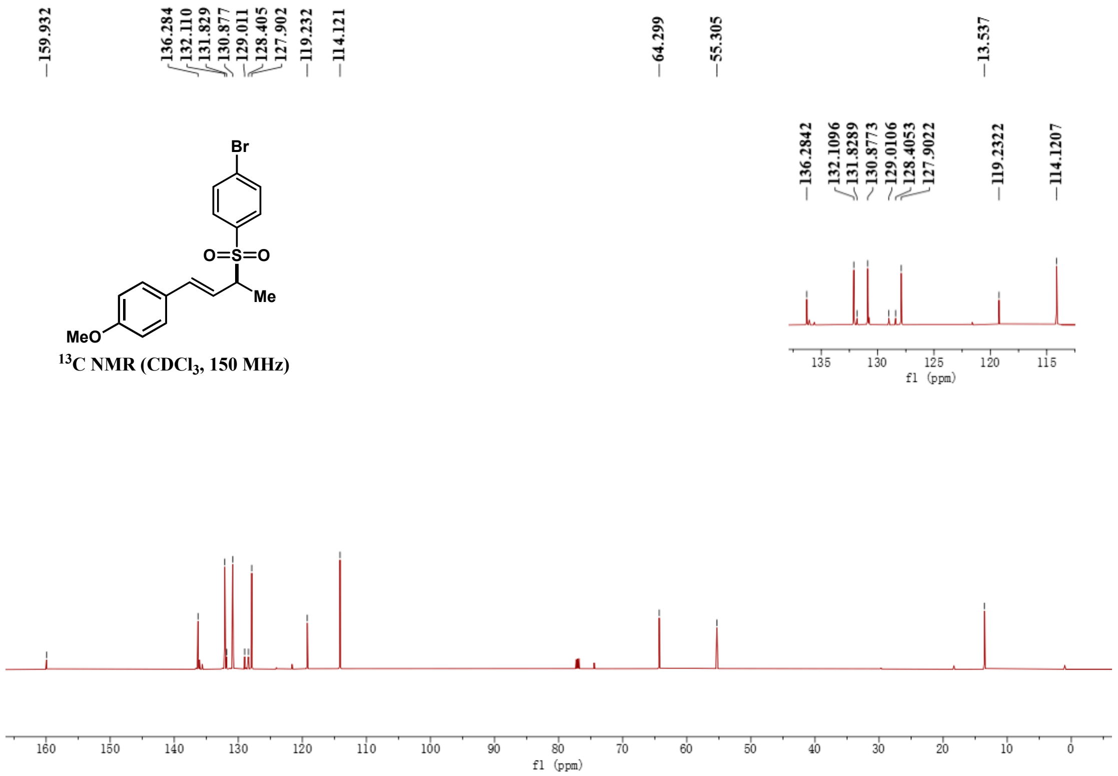

Figure S7. ^{1}H NMR and ^{13}C NMR spectra of **3ae** in $CDCl_3$

3af

^{1}H NMR

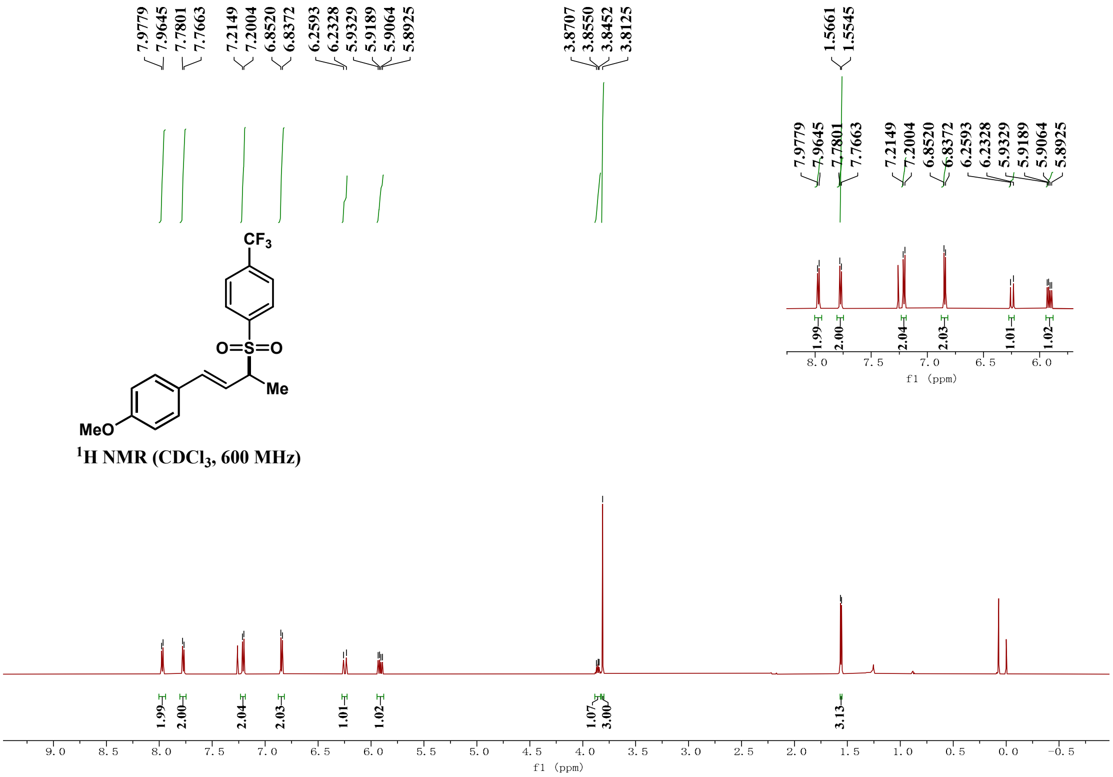

^{13}C NMR

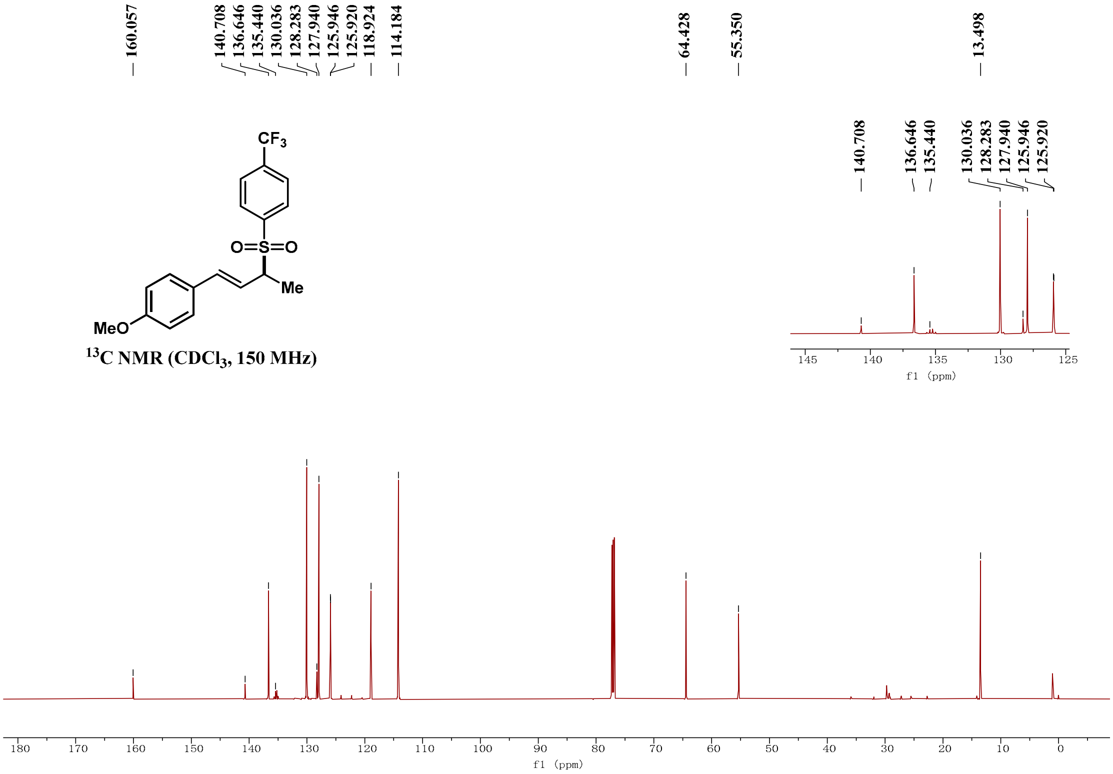

Figure S8. ^{1}H NMR and ^{13}C NMR spectra of **3af** in $CDCl_3$

3ag

^{1}H NMR

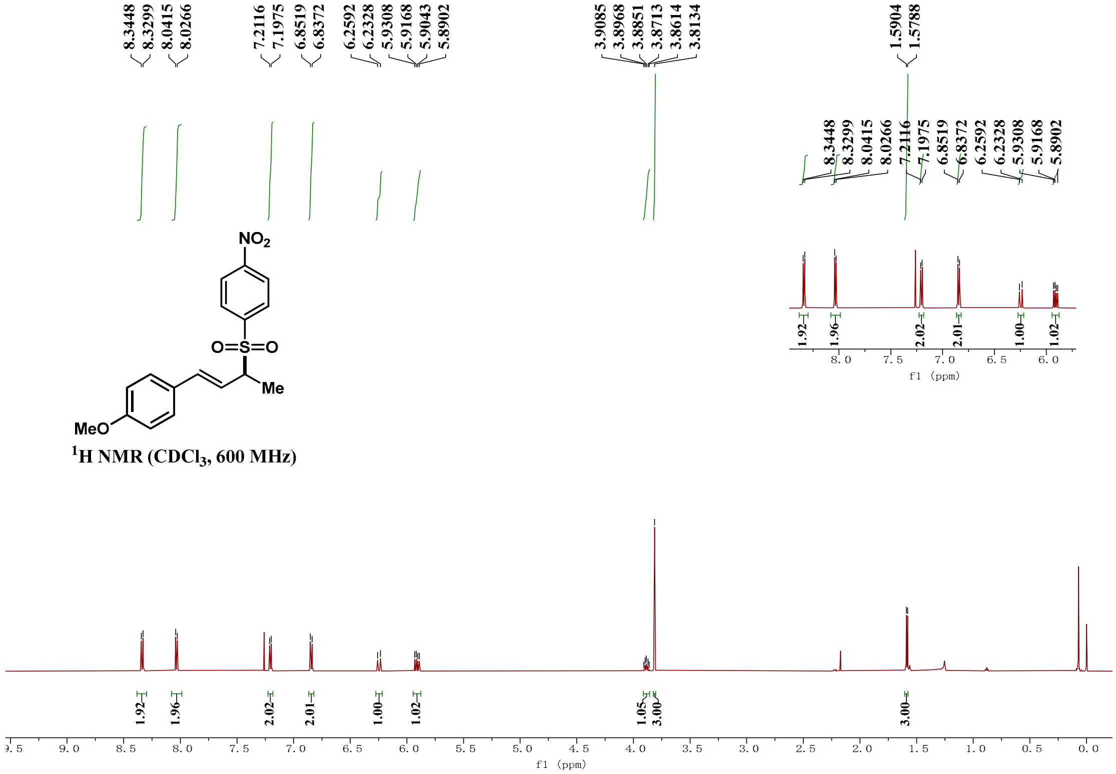

^{13}C NMR

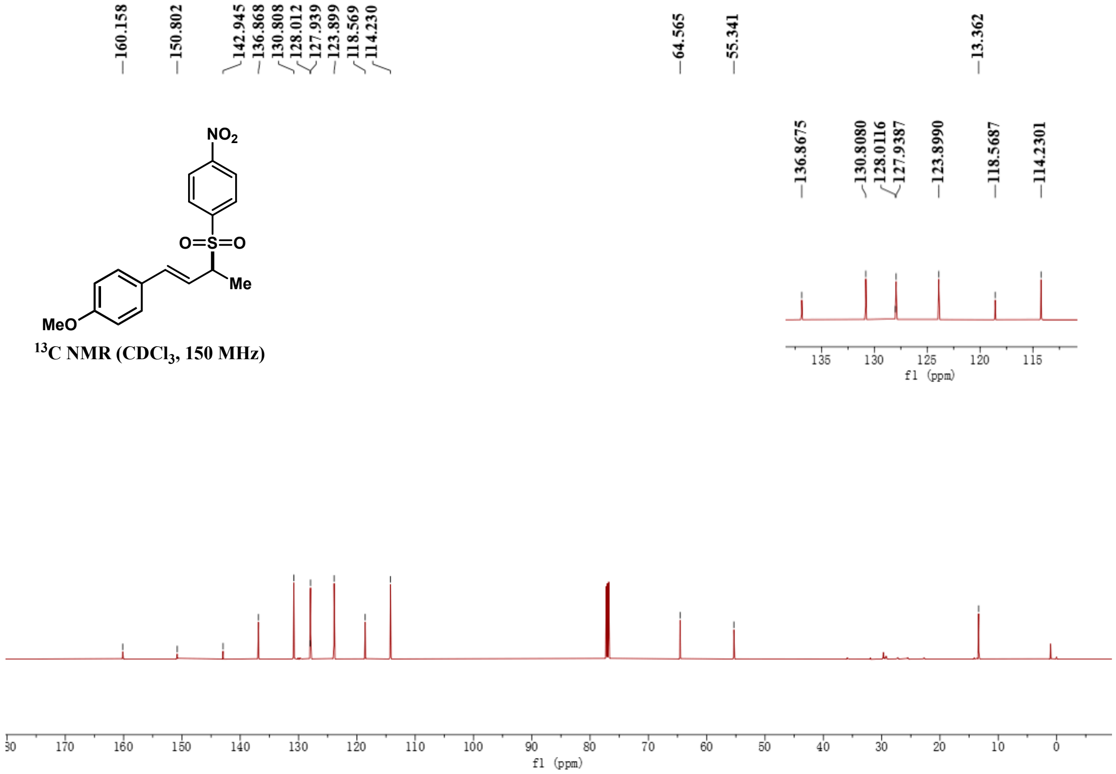

Figure S9. ^{1}H NMR and ^{13}C NMR spectra of **3ag** in $CDCl_3$

3ah

^{1}H NMR

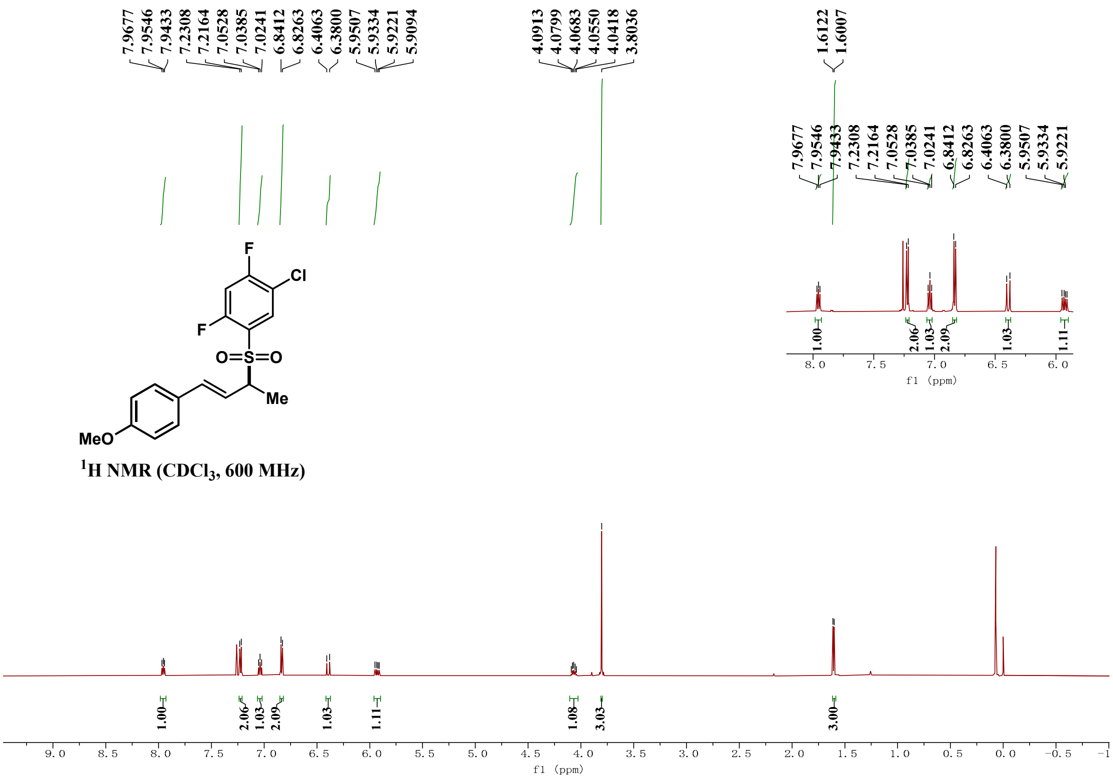

^{13}C NMR

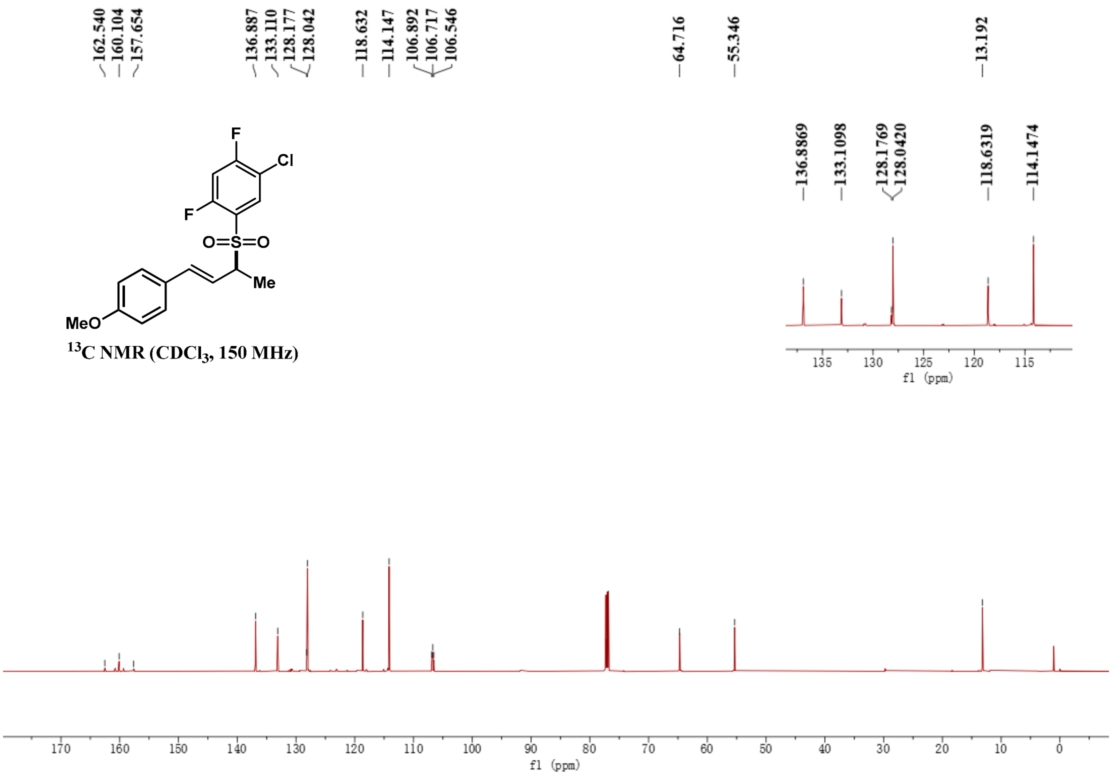

Figure S10. ^{1}H NMR and ^{13}C NMR spectra of **3ah** in $CDCl_3$

3ai

^{1}H NMR

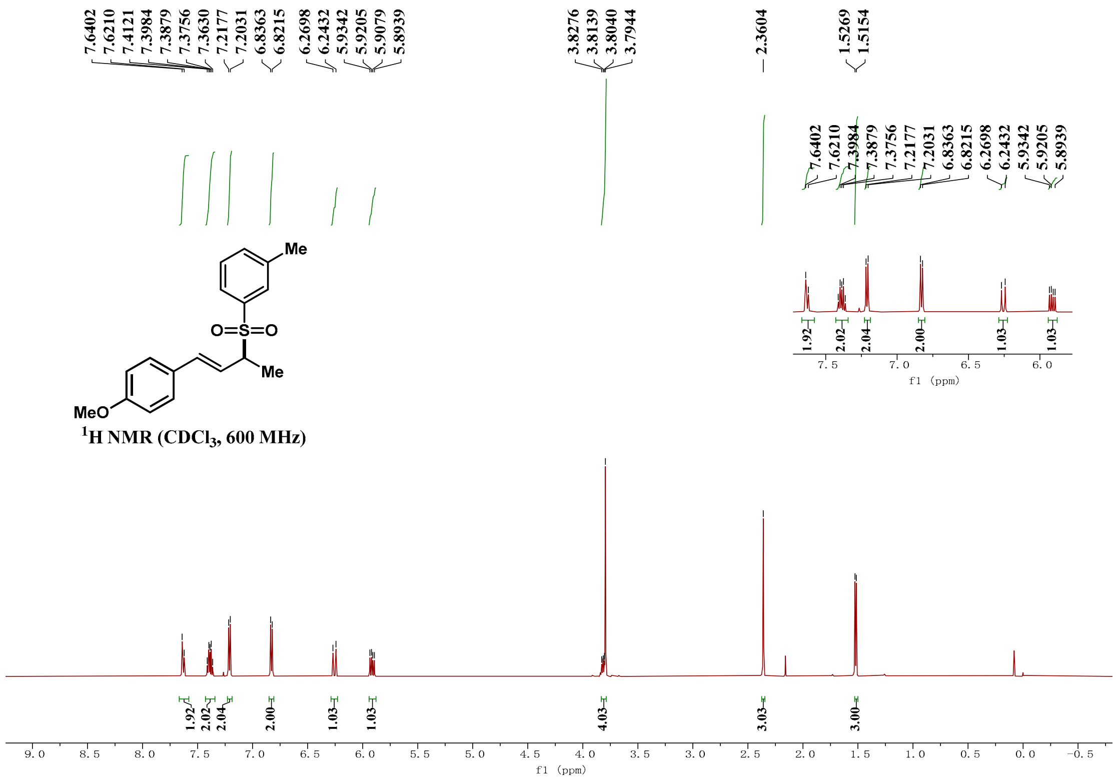

^{13}C NMR

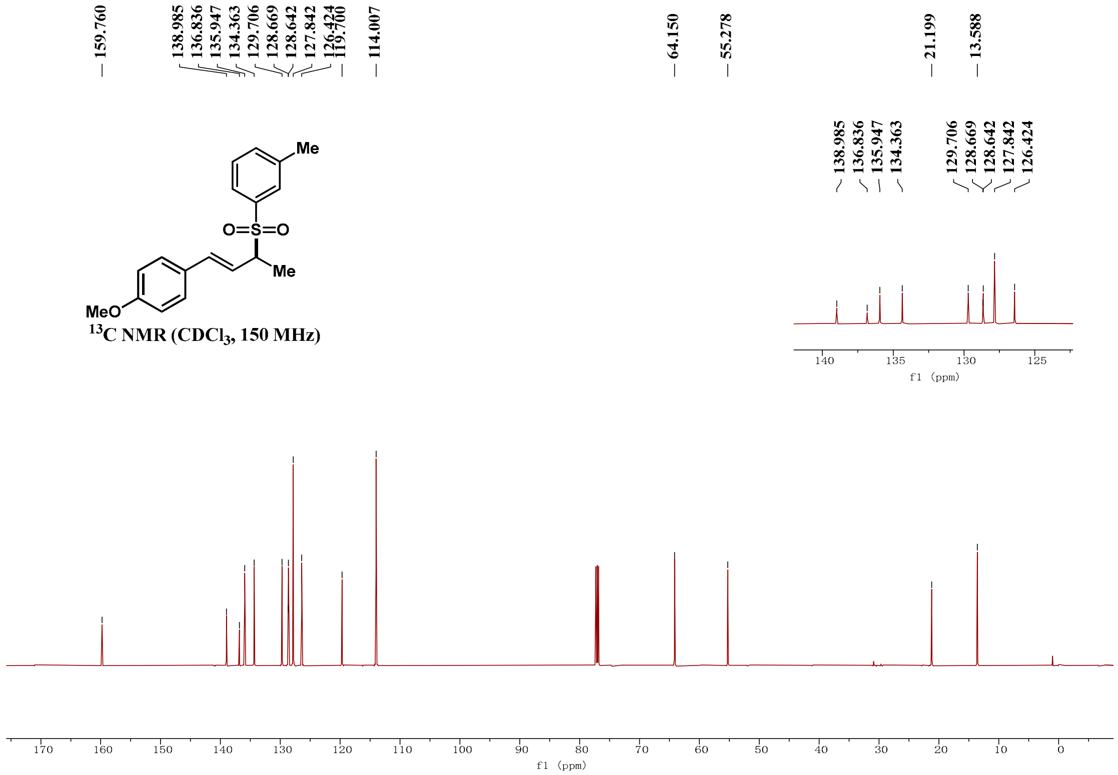

Figure S11. ^{1}H NMR and ^{13}C NMR spectra of **3ai** in $CDCl_3$

3aj

^{1}H NMR

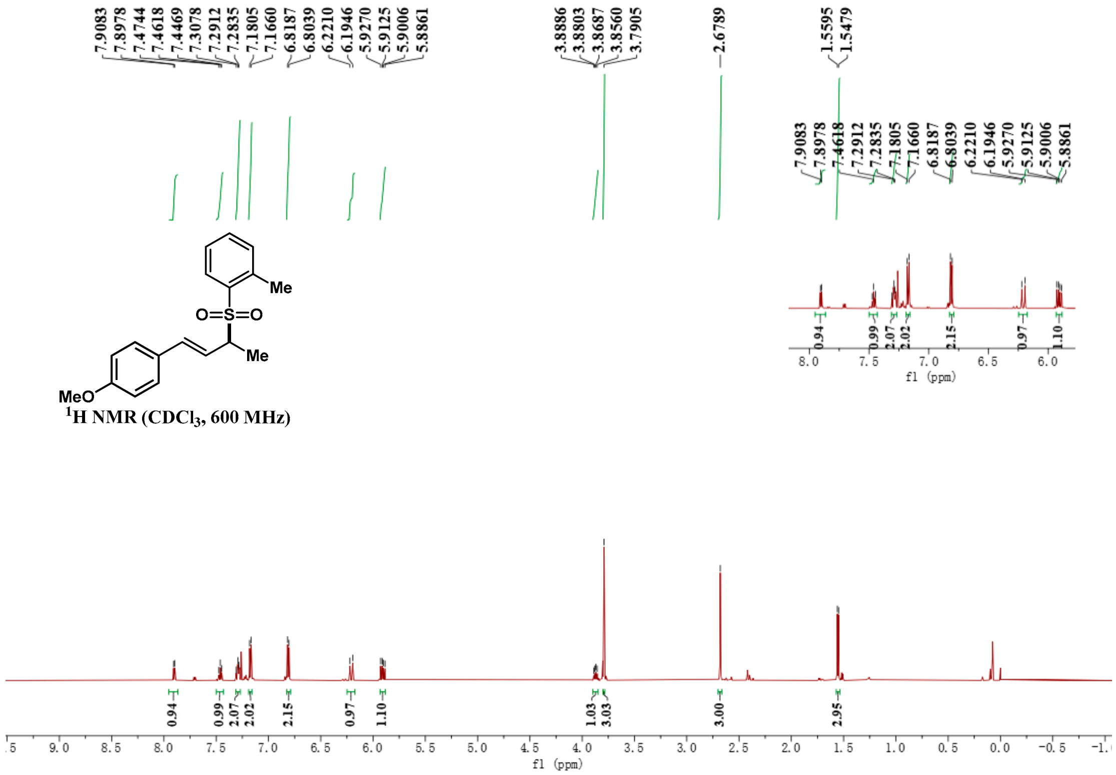

^{13}C NMR

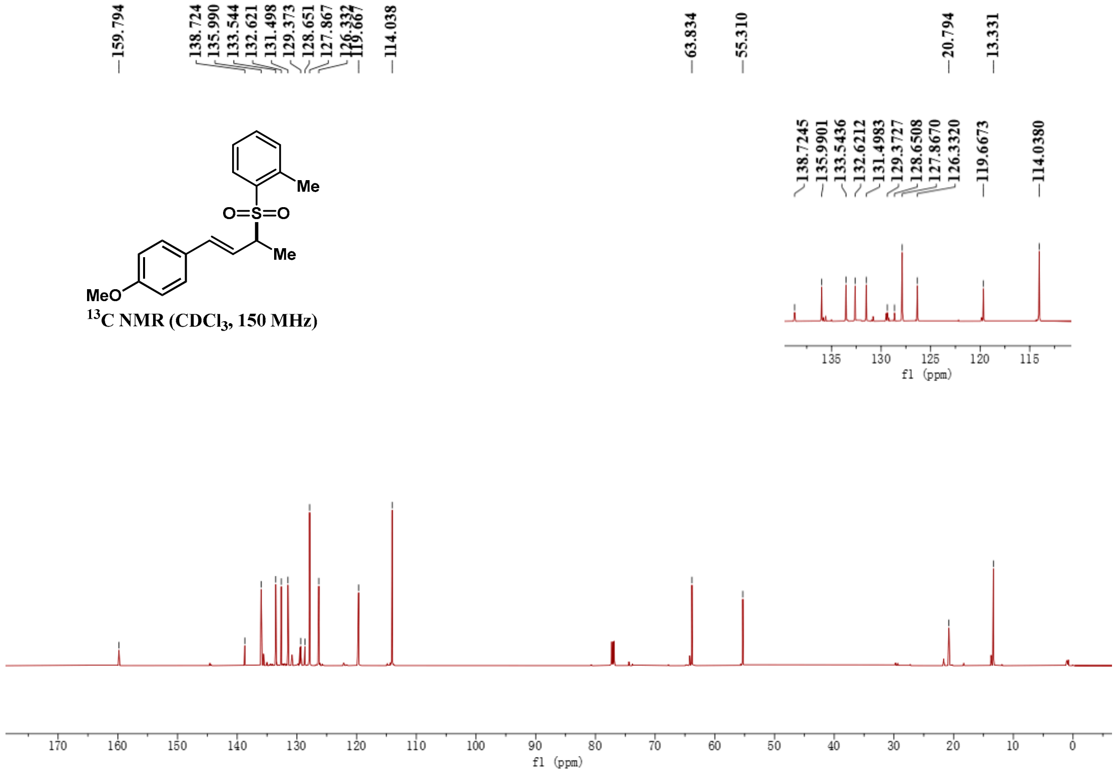

Figure S12. ^{1}H NMR and ^{13}C NMR spectra of **3aj** in $CDCl_3$

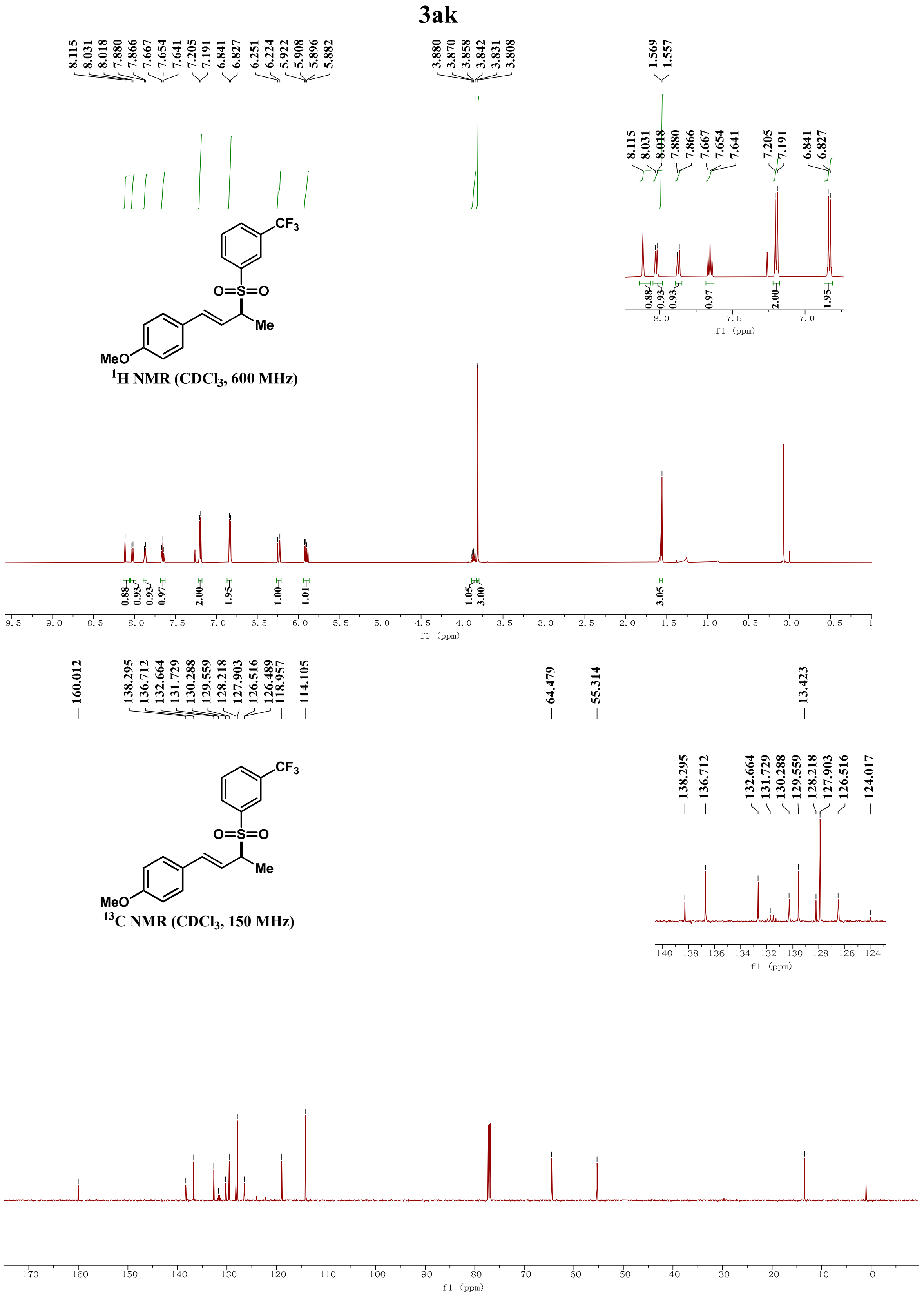

Figure S13. ^{1}H NMR and ^{13}C NMR spectra of **3ak** in $CDCl_3$

3al

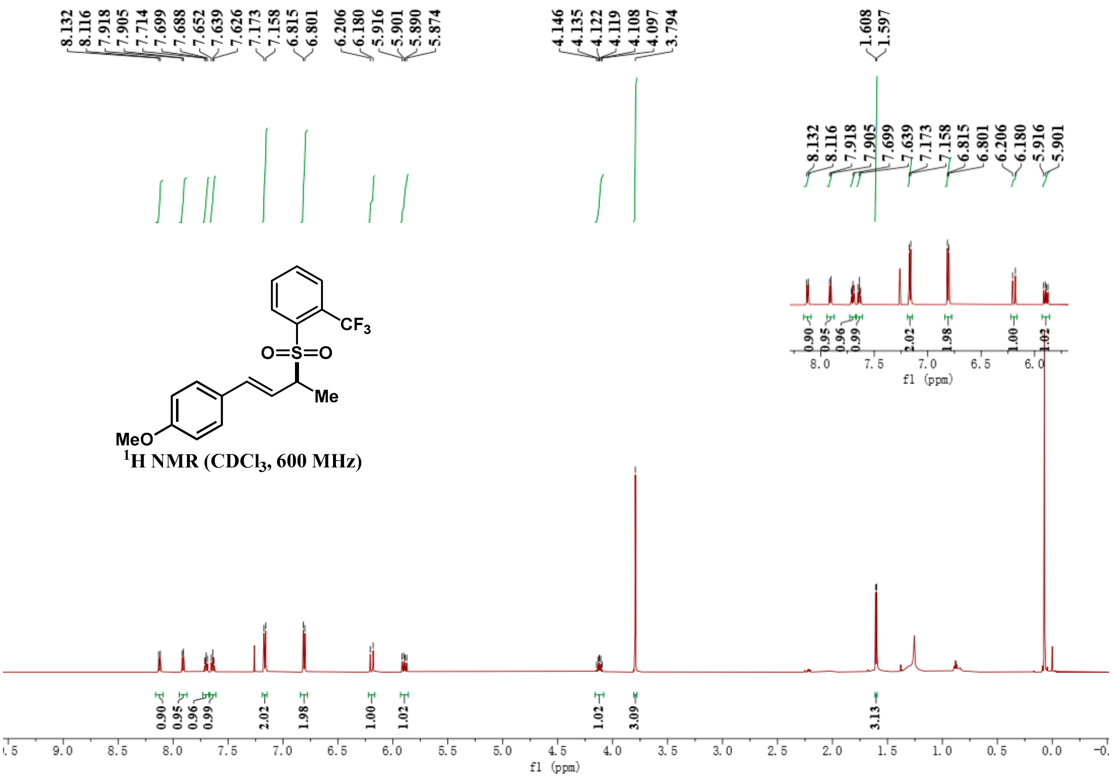

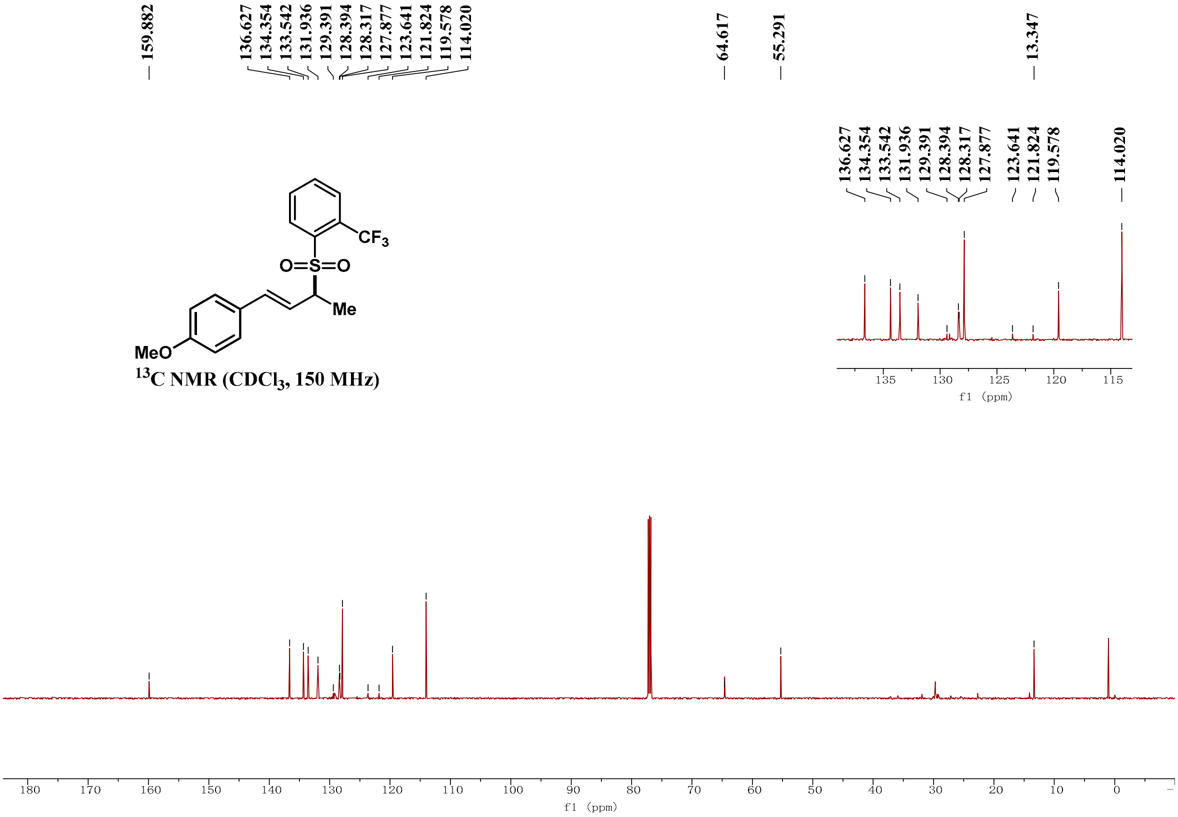

Figure S14. ^{1}H NMR and ^{13}C NMR spectra of **3al** in $CDCl_3$

3am

^{1}H NMR

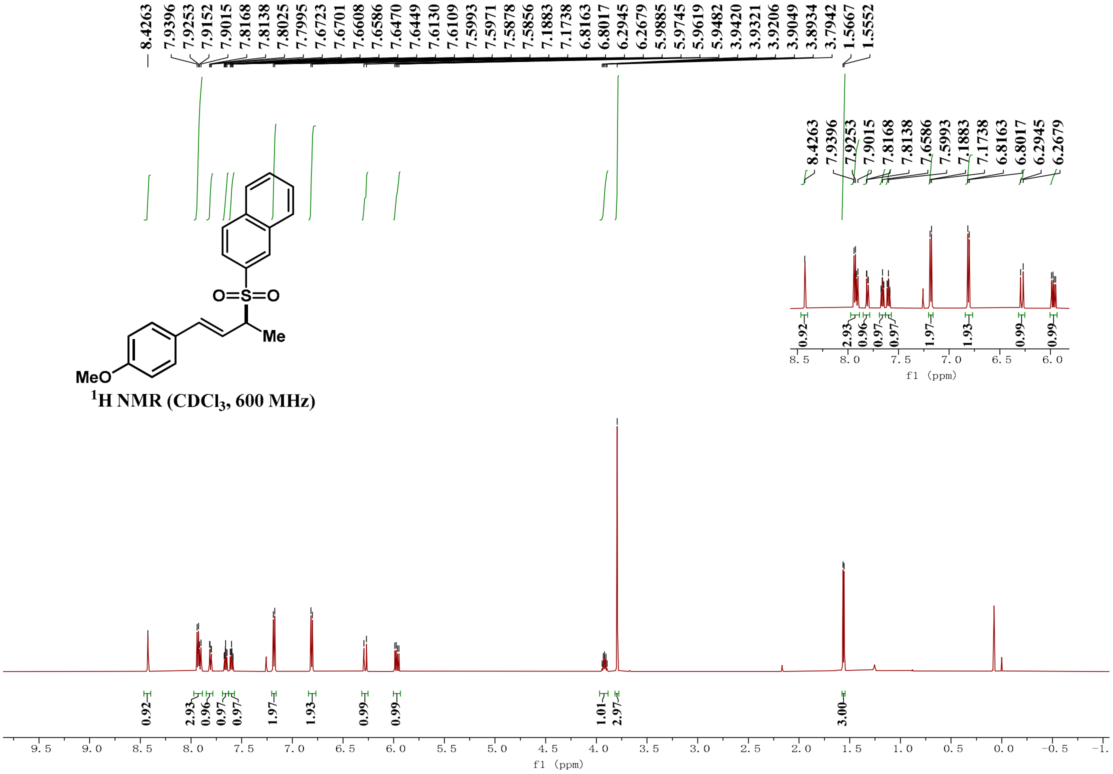

^{13}C NMR

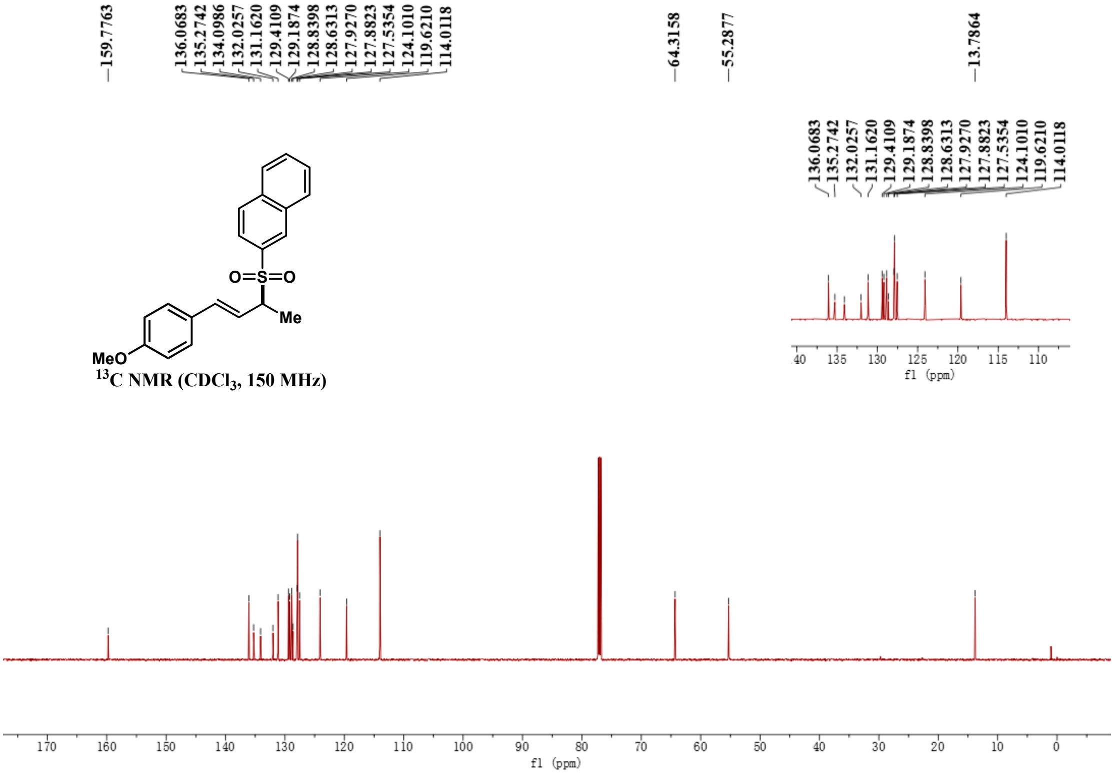

Figure S15. ^{1}H NMR and ^{13}C NMR spectra of **3am** in CDCl$_3$

3an

^{1}H NMR

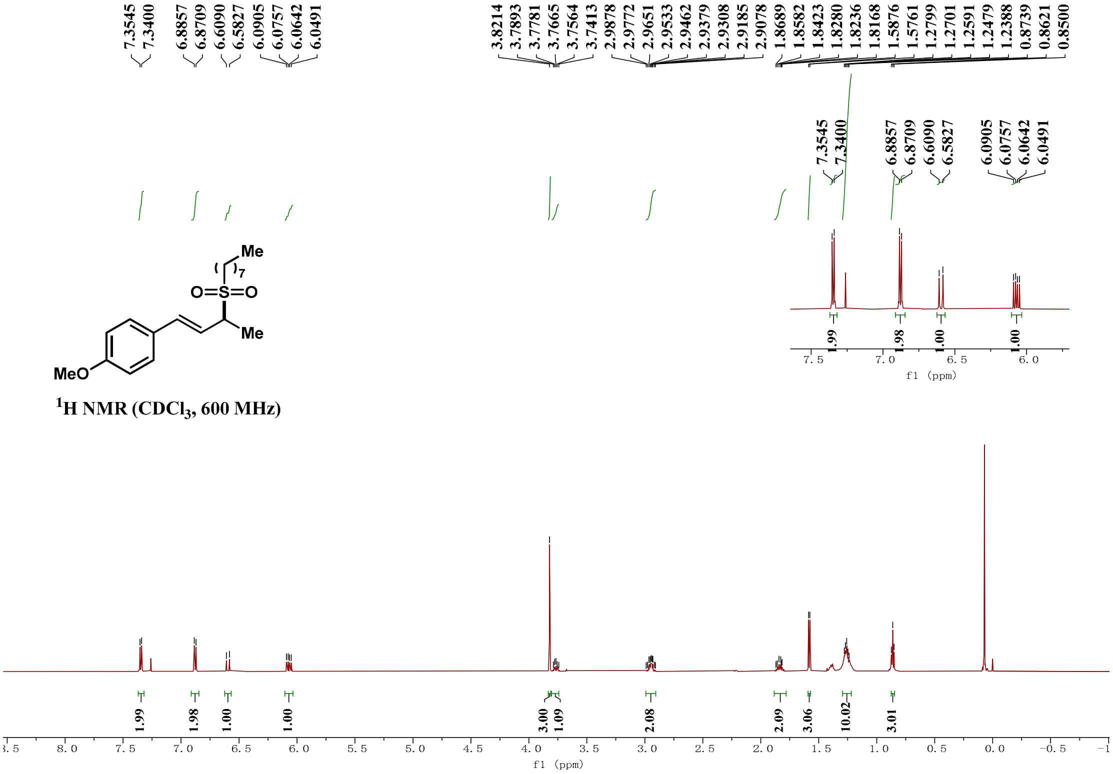

^{13}C NMR

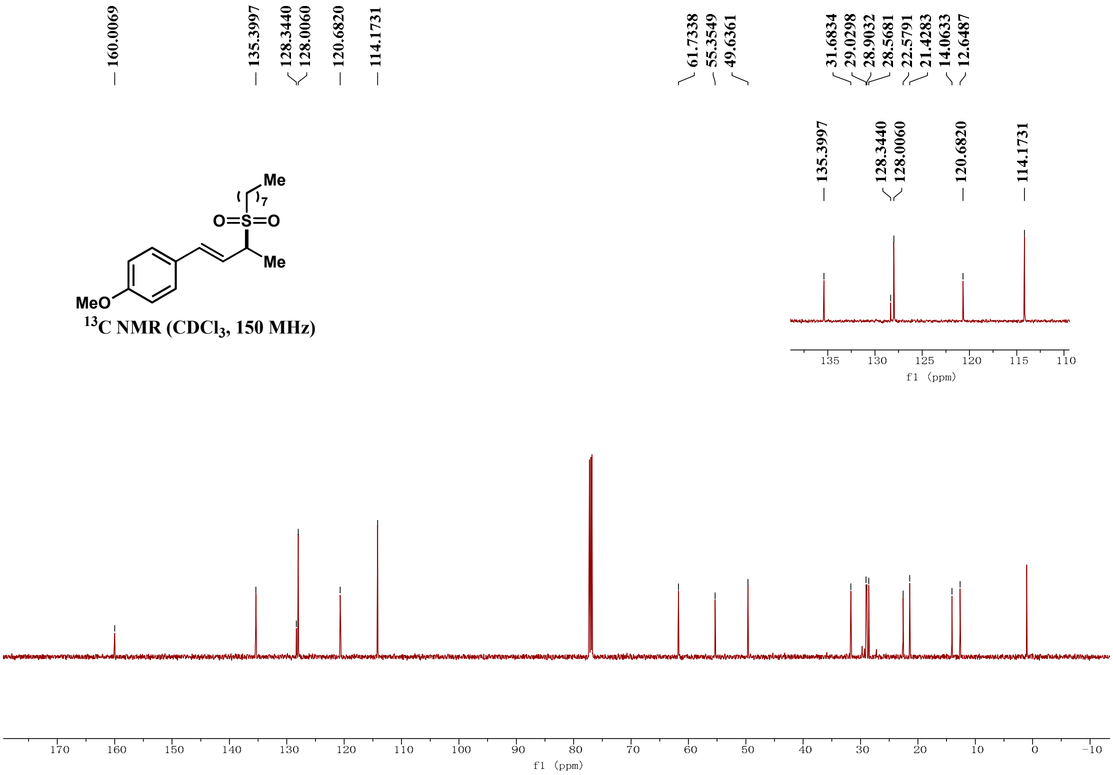

Figure S16. ^{1}H NMR and ^{13}C NMR spectra of **3an** in $CDCl_3$

3ba

^{1}H NMR

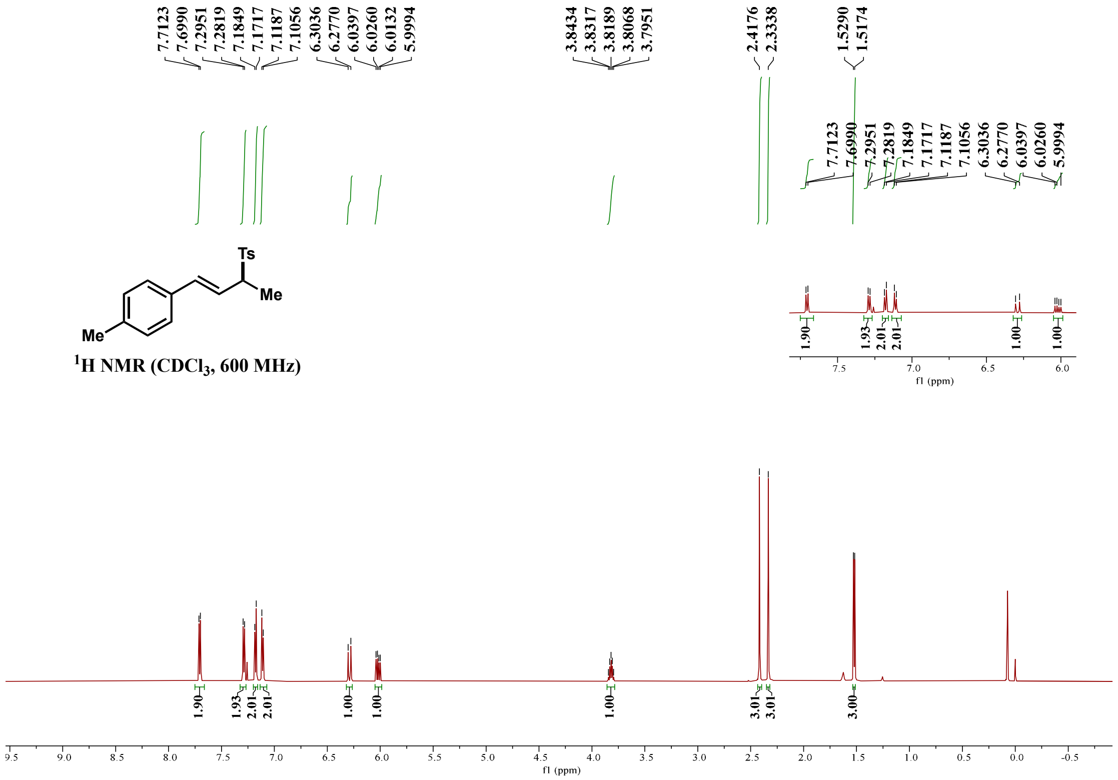

^{13}C NMR

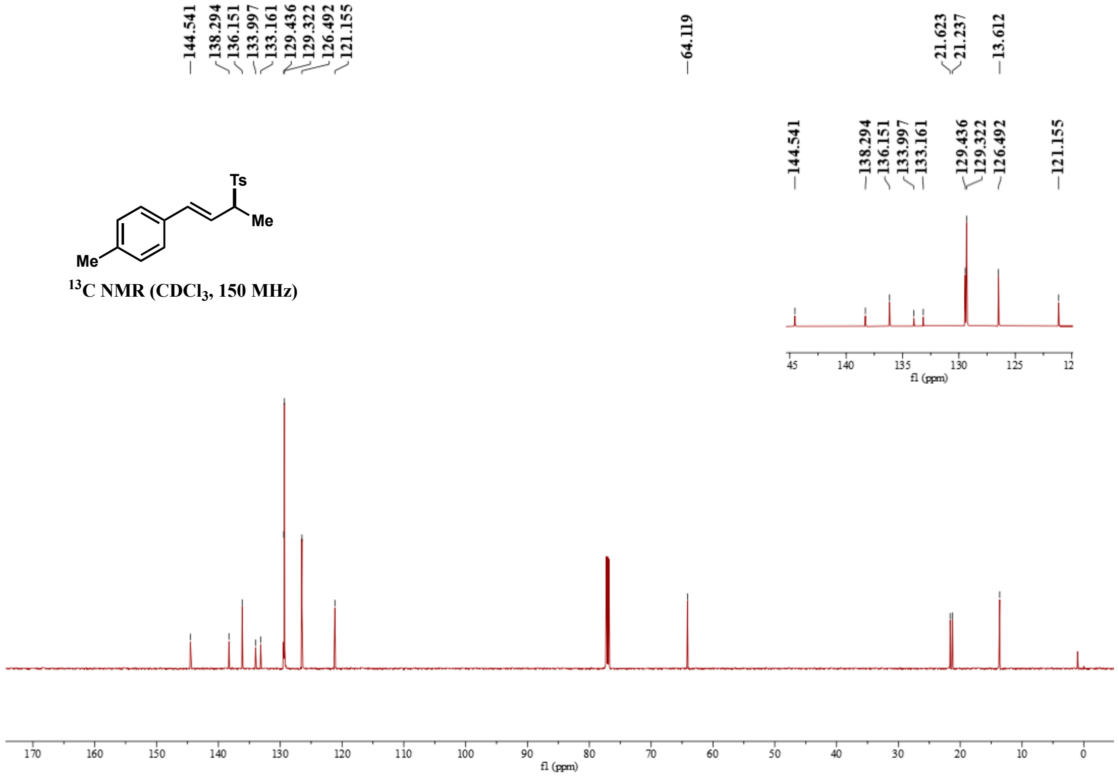

Figure S17. ^{1}H NMR and ^{13}C NMR spectra of **3ba** in $CDCl_3$

3ca

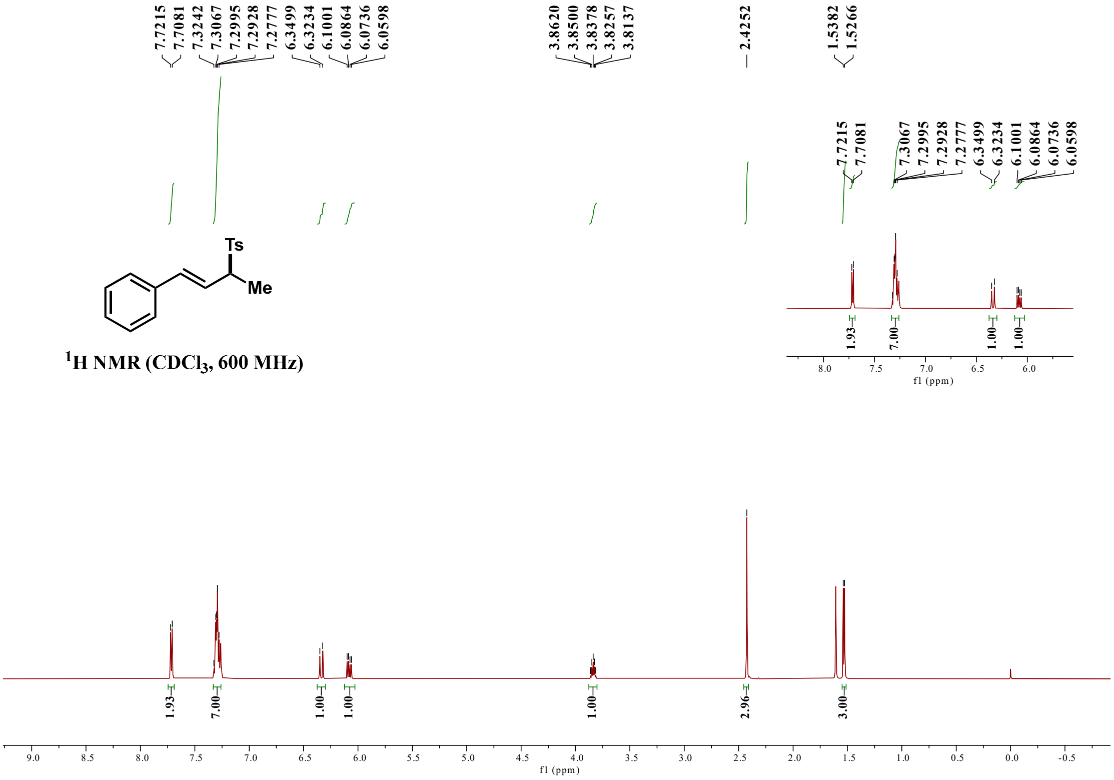

^{13}C NMR

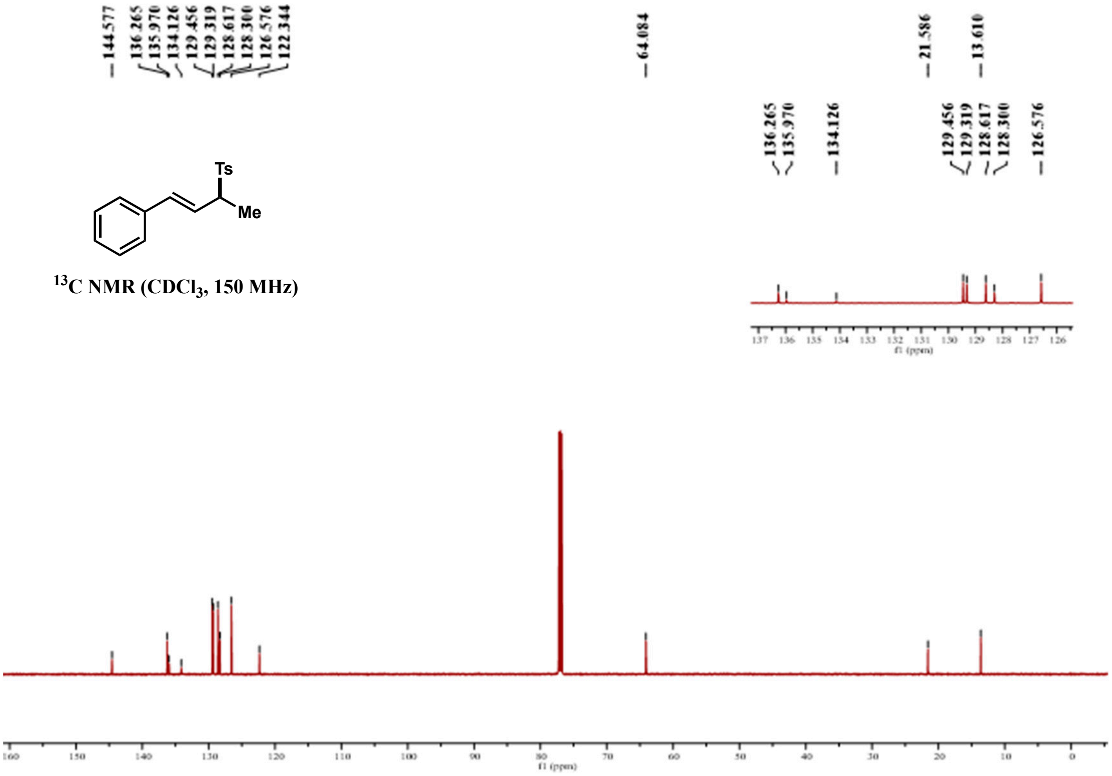

Figure S18. ^{1}H NMR and ^{13}C NMR spectra of **3ca** in $CDCl_3$

3da

^{1}H NMR

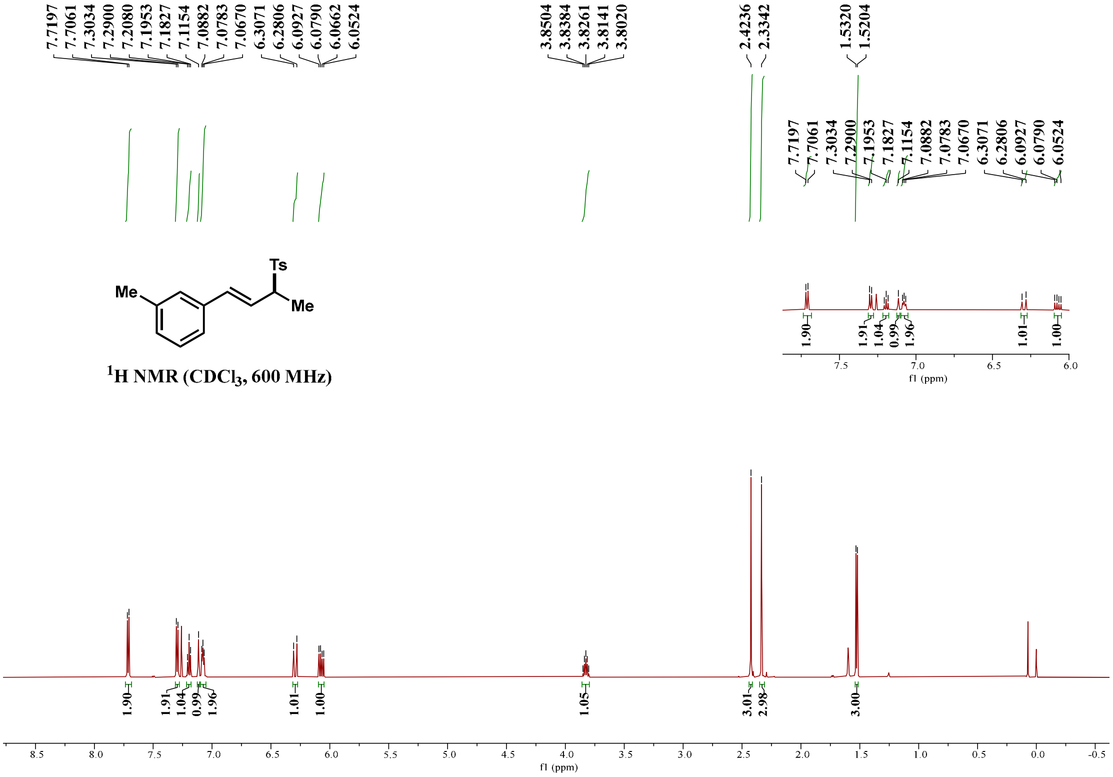

^{13}C NMR

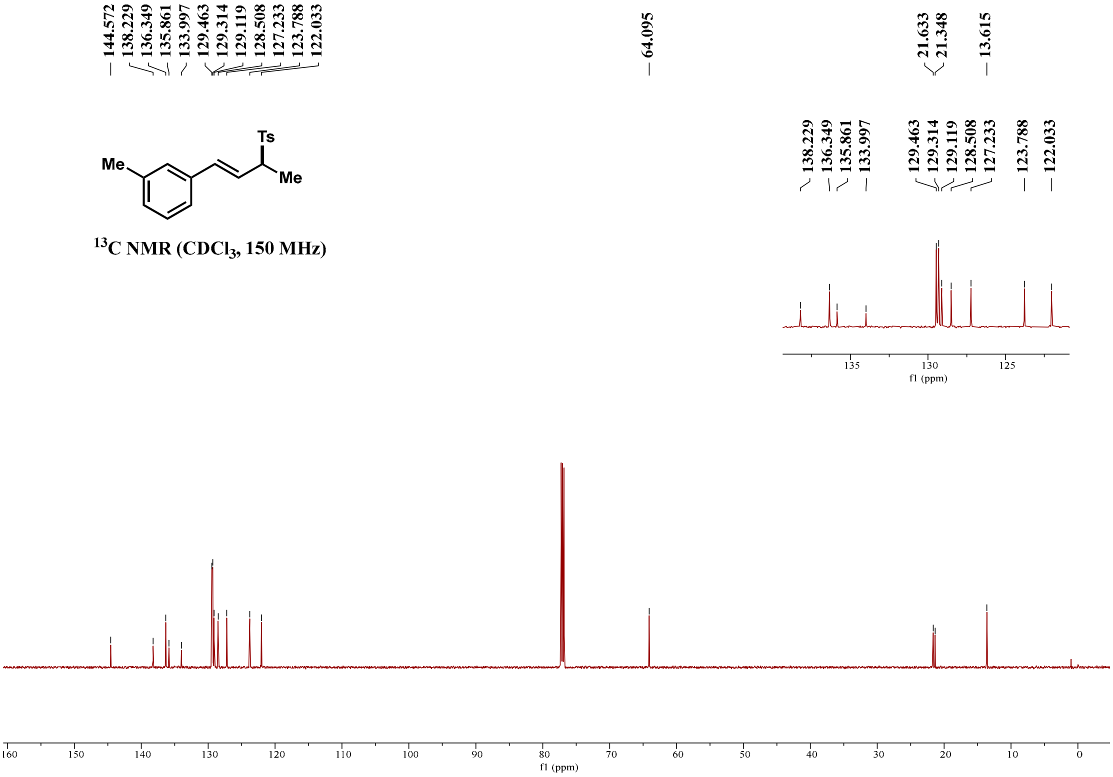

Figure S19. ^{1}H NMR and ^{13}C NMR spectra of **3da** in $CDCl_3$

3ea

^{1}H NMR

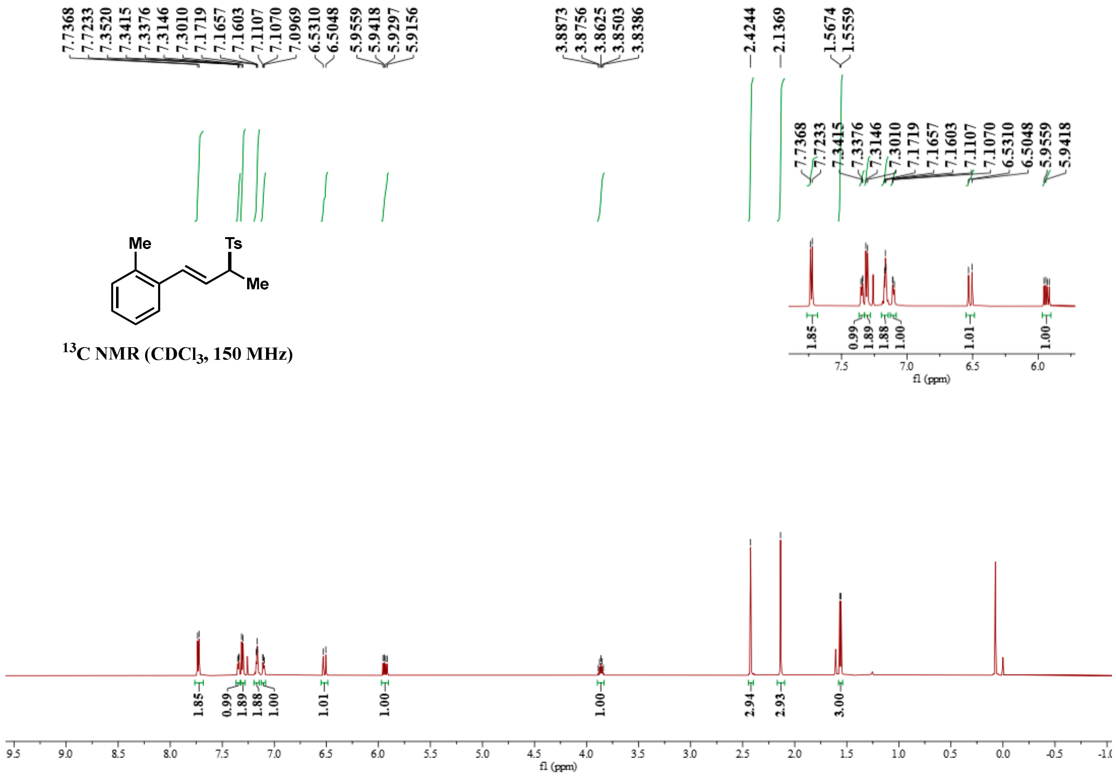

^{13}C NMR

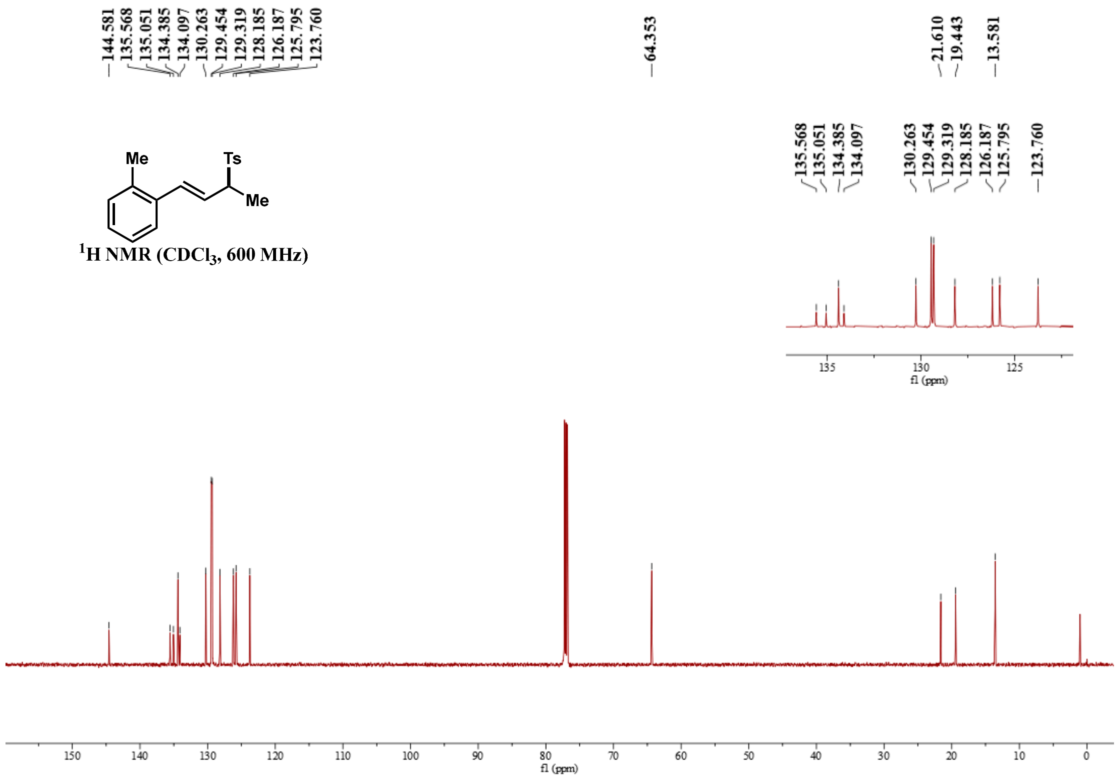

Figure S20. ^{1}H NMR and ^{13}C NMR spectra of **3ea** in $CDCl_3$

3fa

^{1}H NMR

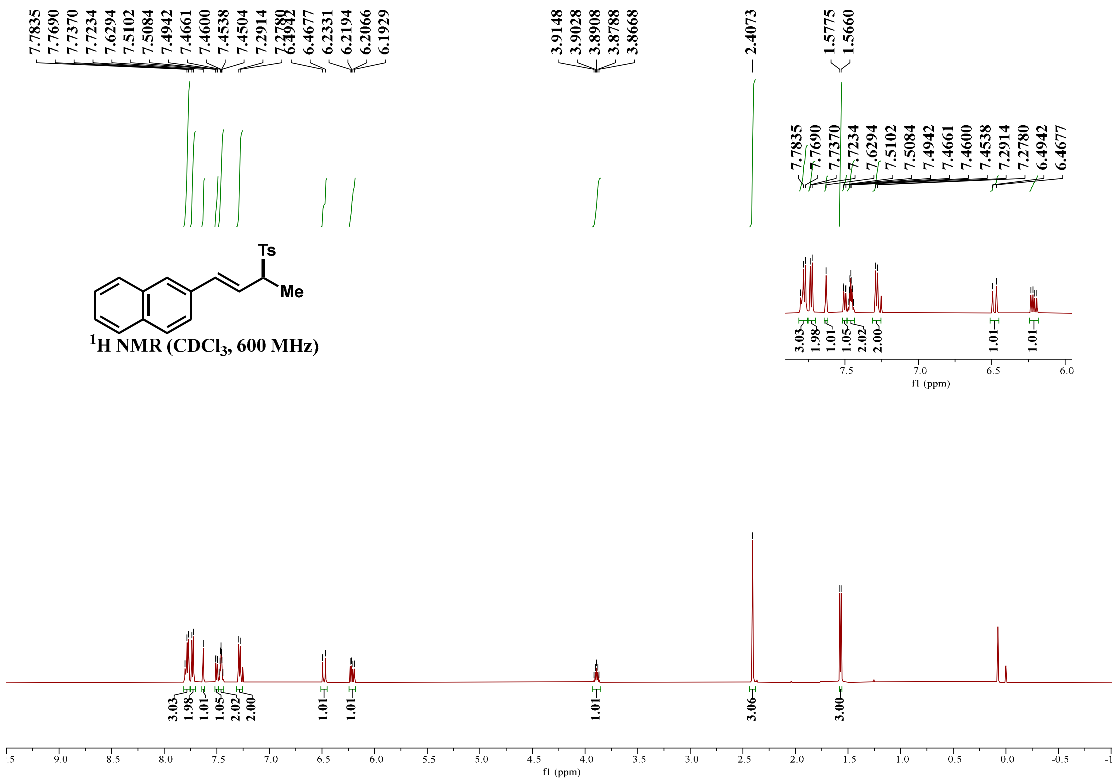

^{13}C NMR

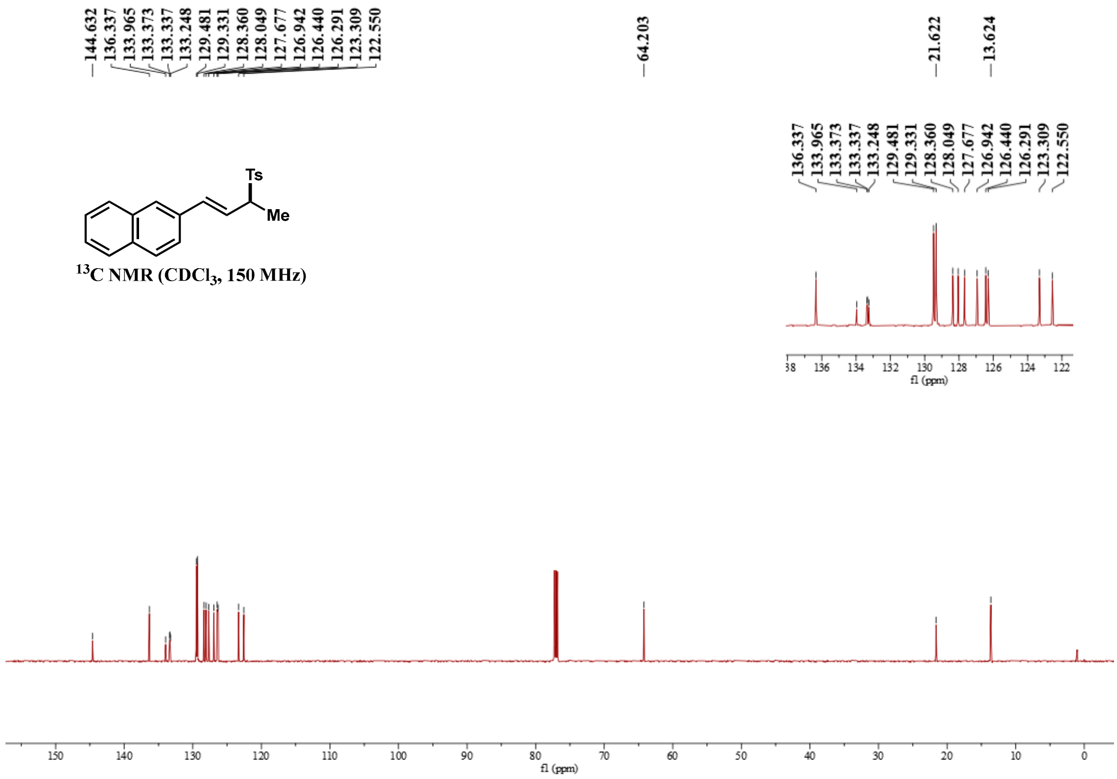

Figure S21. ^{1}H NMR and ^{13}C NMR spectra of **3fa** in $CDCl_3$

3ga

^{1}H NMR

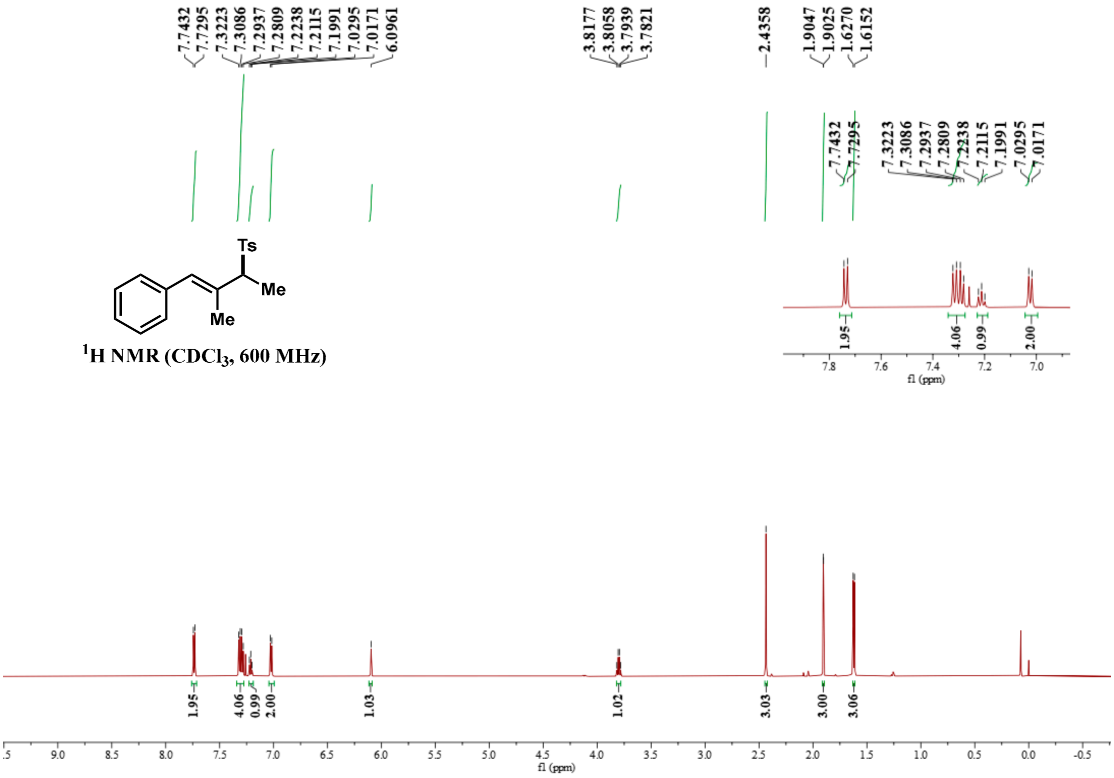

^{13}C NMR

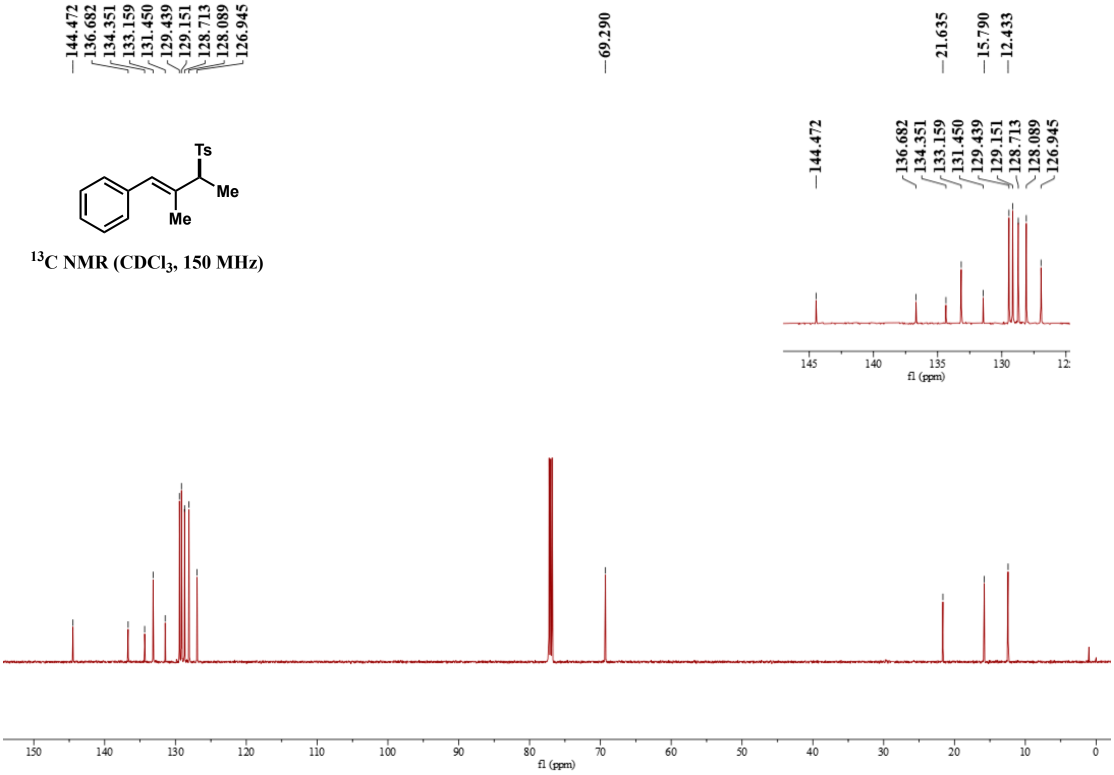

Figure S22. ^{1}H NMR and ^{13}C NMR spectra of **3ga** in $CDCl_3$

3ha

^{1}H NMR

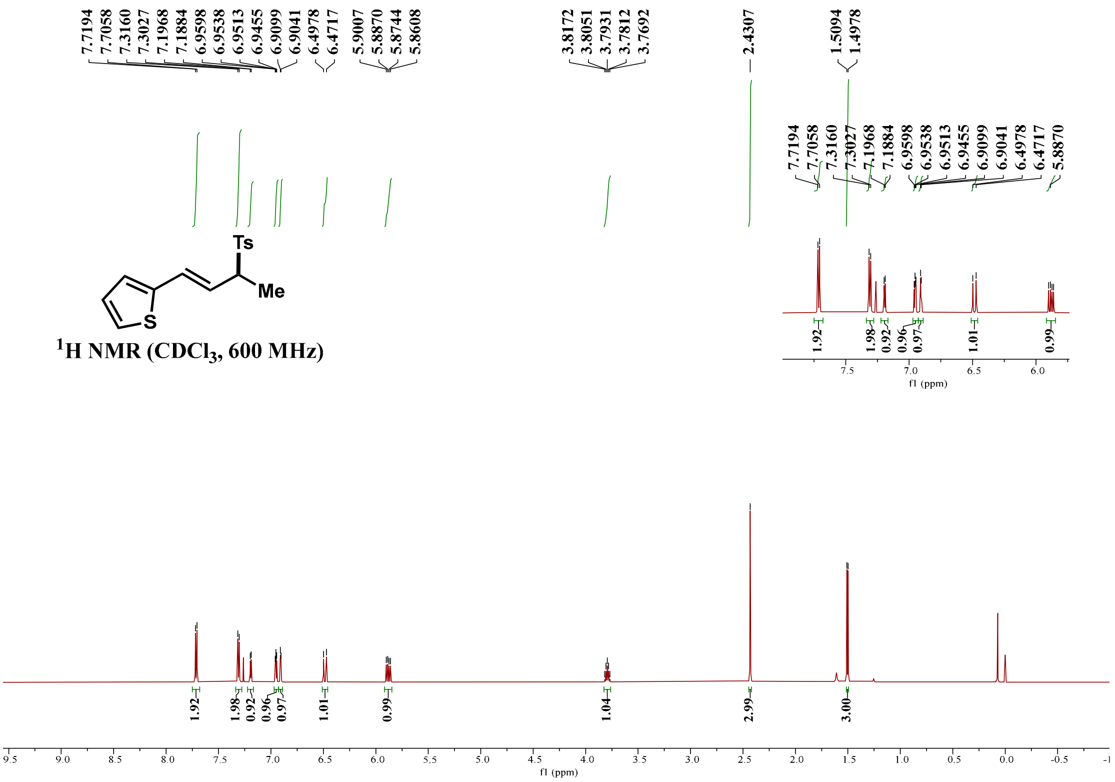

^{13}C NMR

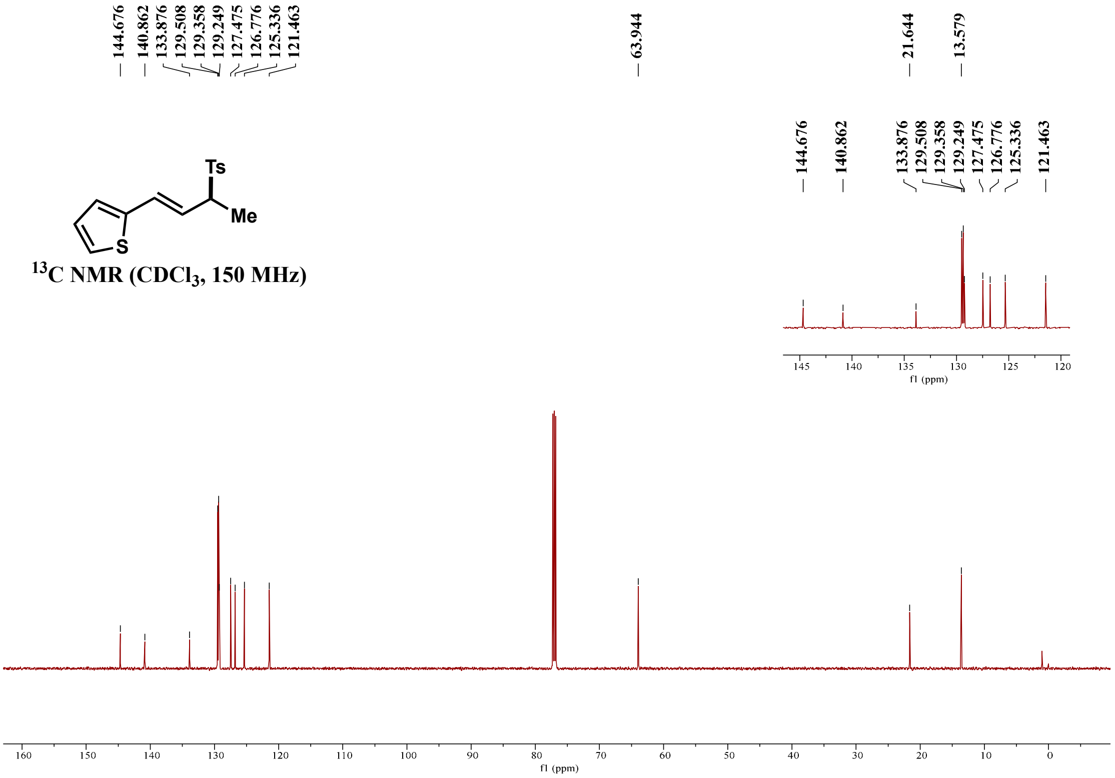

Figure S23. ^{1}H NMR and ^{13}C NMR spectra of **3ha** in $CDCl_3$

3ia

^{1}H NMR

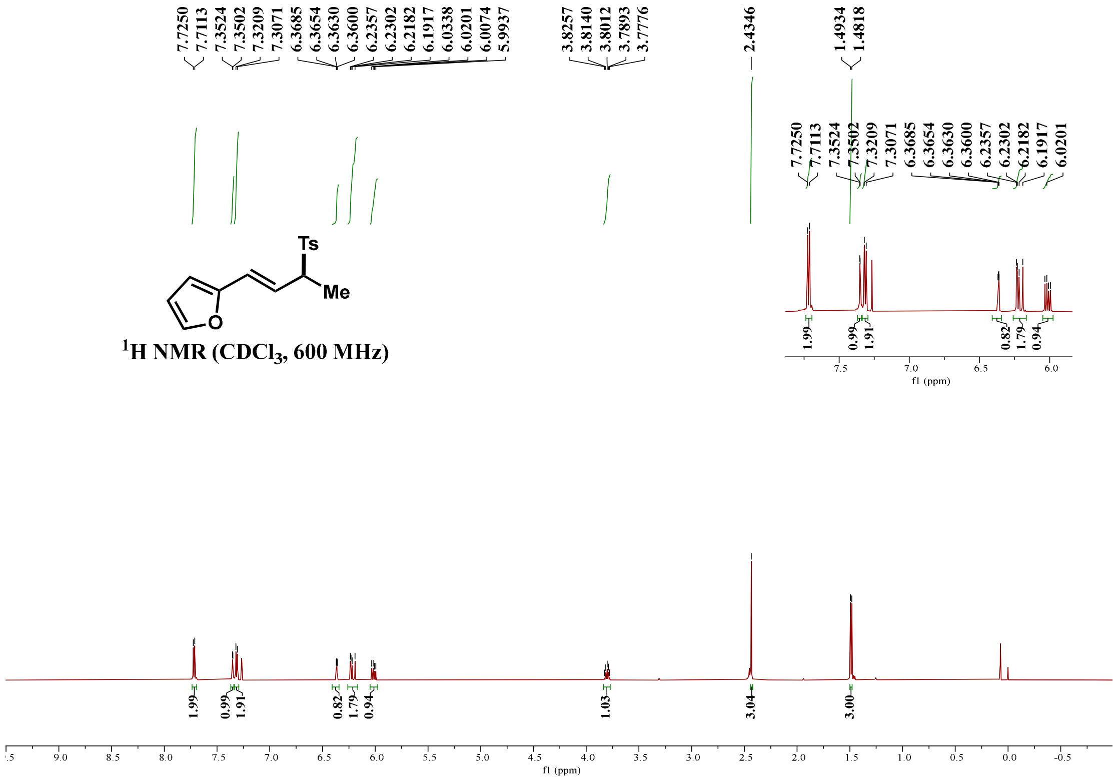

^{13}C NMR

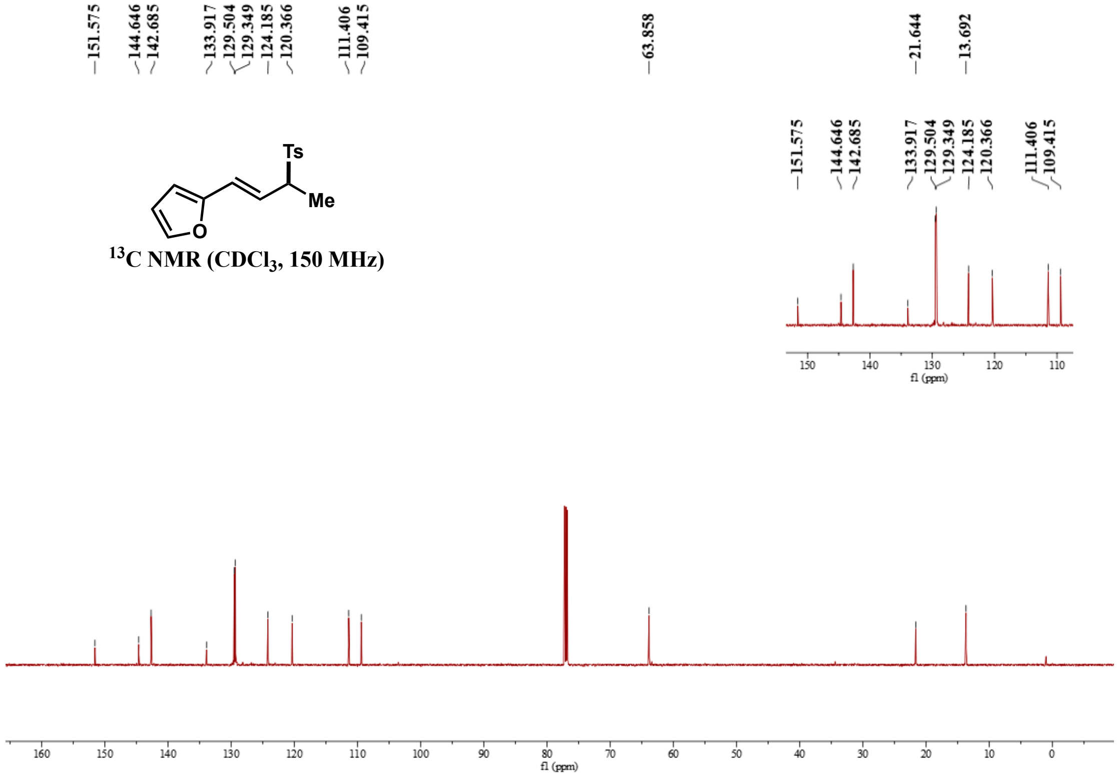

Figure S24. ^{1}H NMR and ^{13}C NMR spectra of **3ia** in $CDCl_3$

3ja

^{1}H NMR

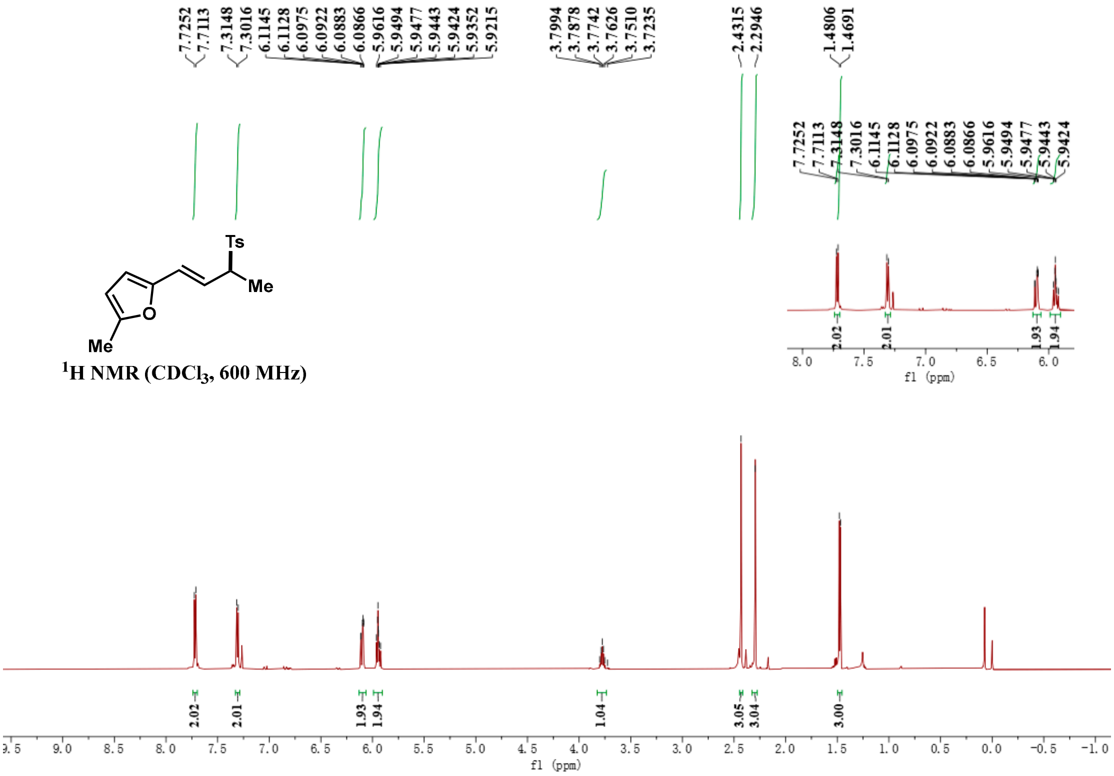

^{13}C NMR

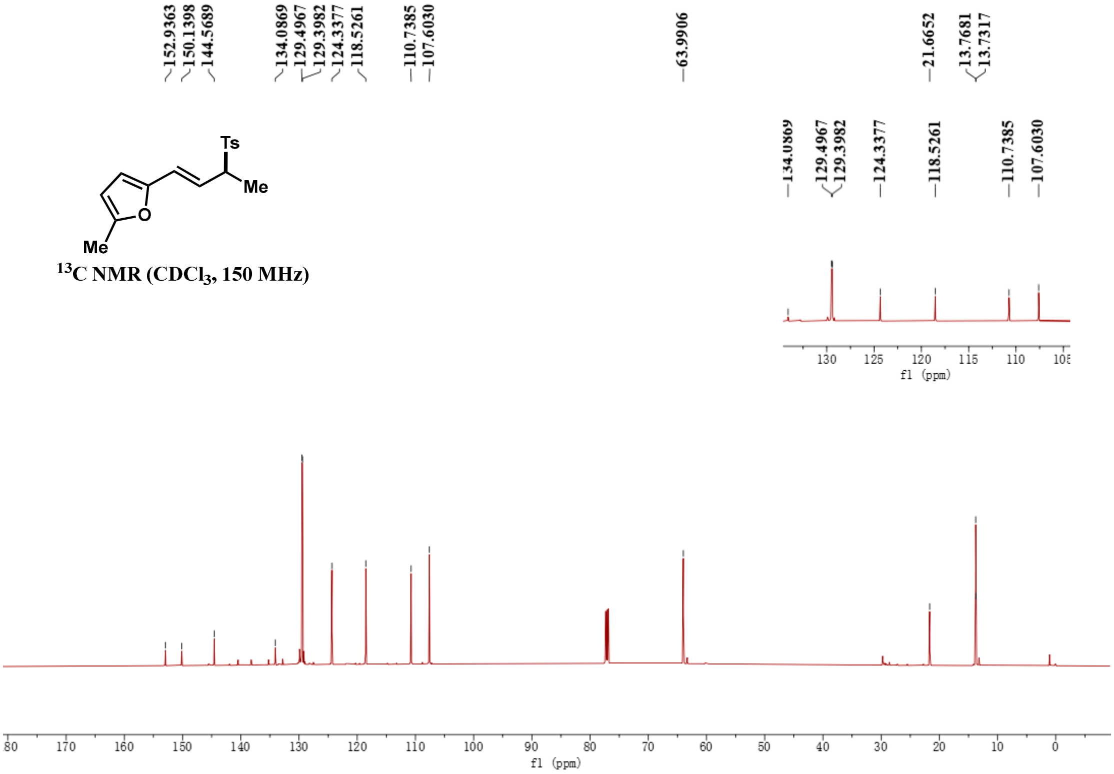

Figure S25. ^{1}H NMR and ^{13}C NMR spectra of **3ja** in $CDCl_3$

4a

^{1}H NMR

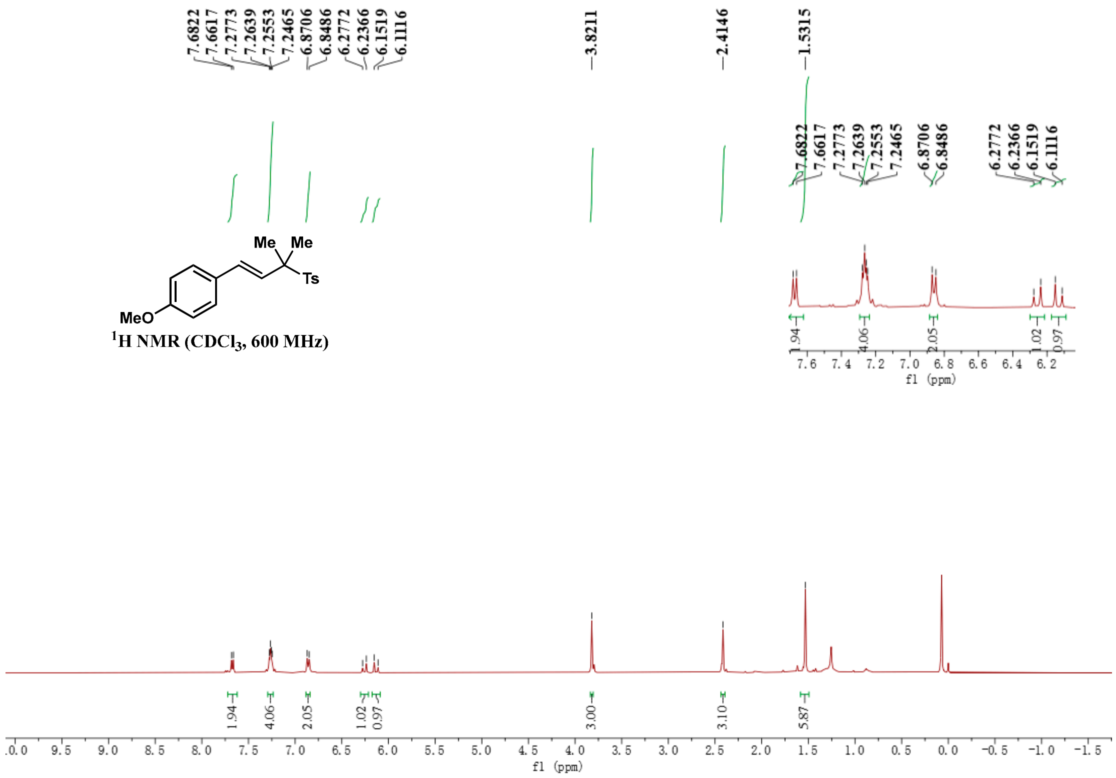

^{13}C NMR

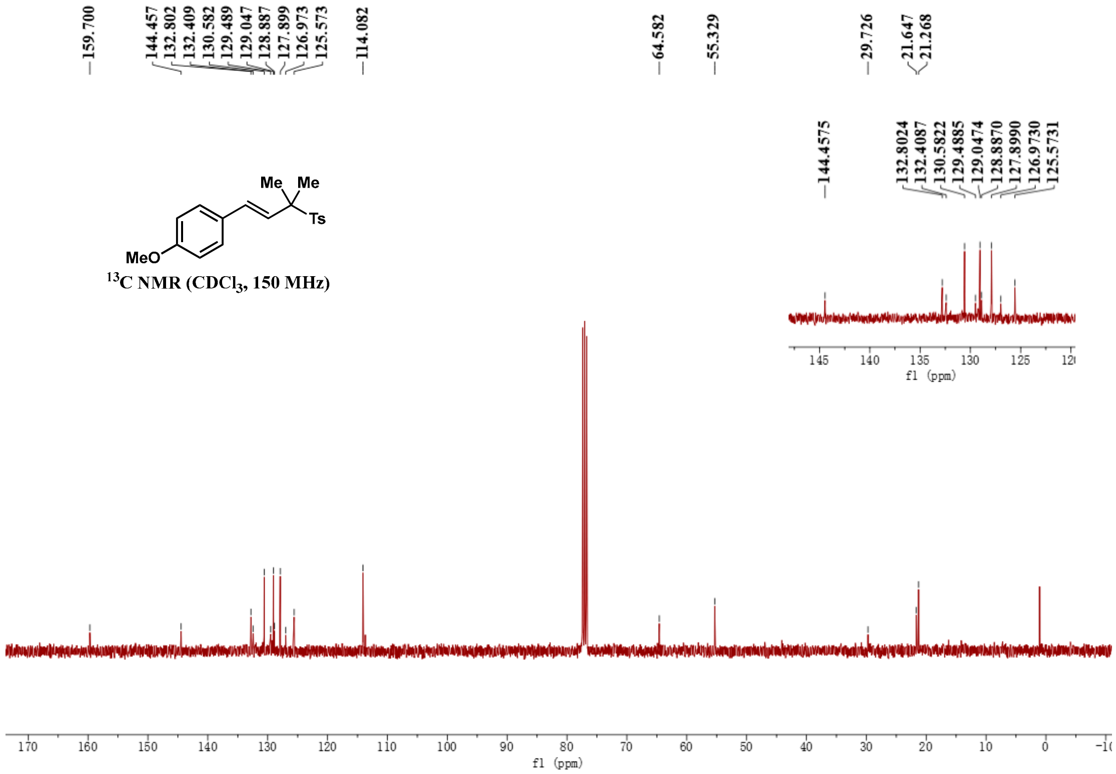

Figure S26. ^{1}H NMR and ^{13}C NMR spectra of **4a** in $CDCl_3$

4b

^{1}H NMR

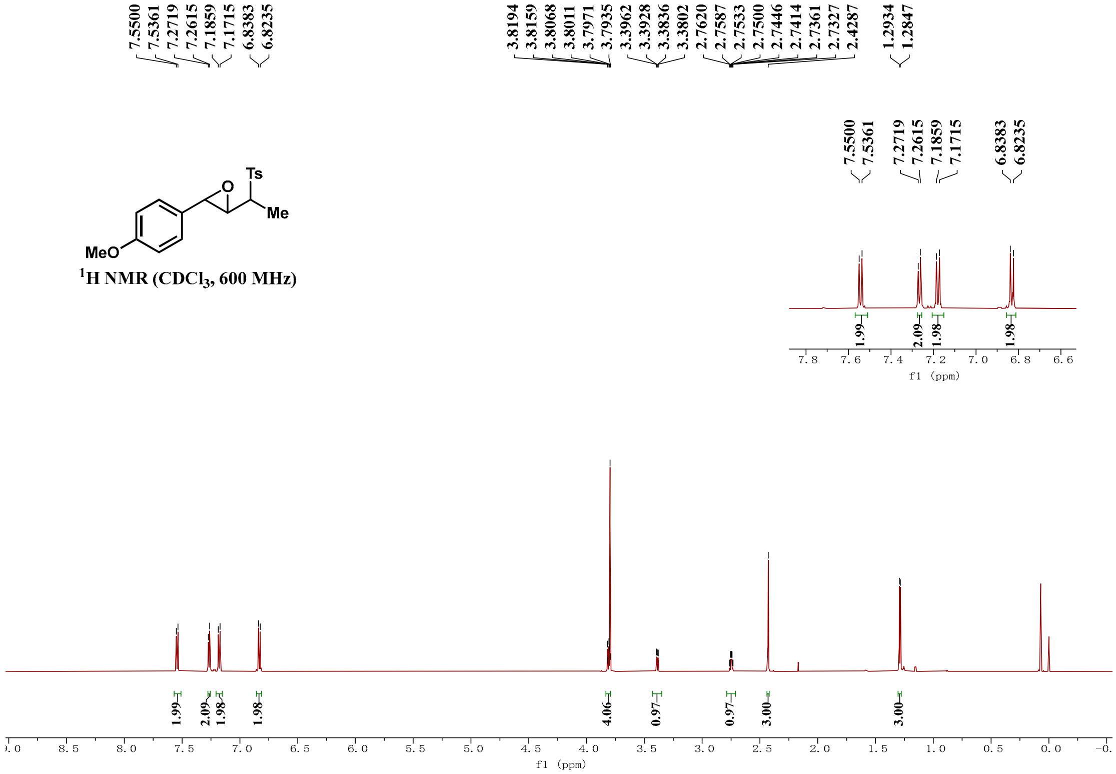

^{13}C NMR

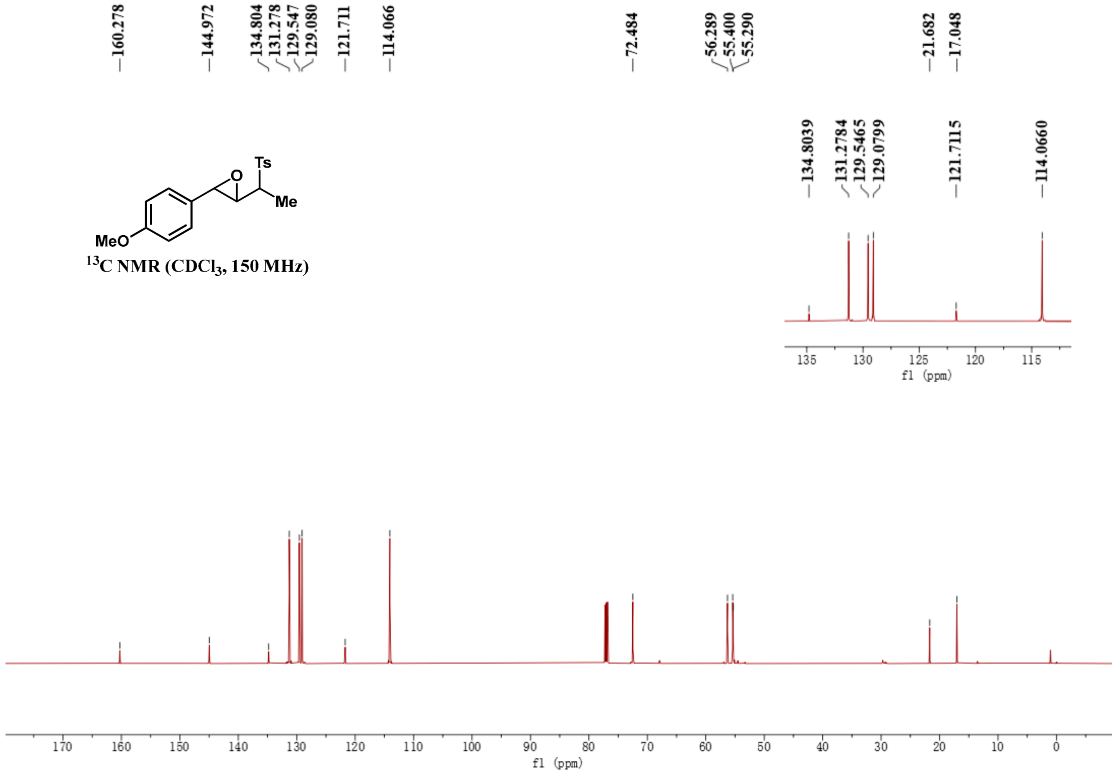

Figure S27. ^{1}H NMR and ^{13}C NMR spectra of **4b** in $CDCl_3$

5. References

[1] (a) F. Puenner, A. Schmidt and G. Hilt, Angew. Chem. Int. Ed. 51 (2012) 1270-1273;
(b) T. Preuß, W. Saak and S. Doye, Chem–Eur. J. 19 (2013) 3833-3837.

[2] P. Qian, Y. Deng, H. Mei, J. Han, J. Zhou and Y. Pan, Org. Lett. 19 (2017) 4798-4801.

[3] Z. T. Ariki, Y. Maekawa, M. Nambo and C. M. Crudden, J. Am. Chem. Soc. 140 (2018) 78-81.

[4] Q. Zhang, D. Dong and W. Zi, J. Am. Chem. Soc. 142 (2020) 15860-15869.
